# GPCR structure, function, drug discovery and crystallography: report from Academia-Industry International Conference (UK Royal Society) Chicheley Hall, 1–2 September 2014

**DOI:** 10.1007/s00210-015-1111-8

**Published:** 2015-03-14

**Authors:** Alexander Heifetz, Gebhard F. X. Schertler, Roland Seifert, Christopher G. Tate, Patrick M. Sexton, Vsevolod V. Gurevich, Daniel Fourmy, Vadim Cherezov, Fiona H. Marshall, R. Ian Storer, Isabel Moraes, Irina G. Tikhonova, Christofer S. Tautermann, Peter Hunt, Tom Ceska, Simon Hodgson, Mike J. Bodkin, Shweta Singh, Richard J. Law, Philip C. Biggin

**Affiliations:** 1 Evotec (UK) Ltd., 114 Innovation Drive, Milton Park, Abingdon, Oxfordshire OX14 4RZ UK; 2 D-BIOL, ETH Zurich Paul Scherrer Institute, Laboratory of Biomolecular Research, LBR OFLC 109, CH-5232 Villigen PSI, Switzerland; 3 Hannover Medical School, Carl-Neuberg-Straße 1, 30625 Hannover, Germany; 4 MRC Laboratory of Molecular Biology, Cambridge Biomedical Campus, Francis Crick Avenue, Cambridge, CB2 0QH UK; 5 Drug Discovery Biology Monash Institute of Pharmaceutical Sciences 381 Royal Parade, Parkville, VIC 3052 Australia; 6 Vanderbilt University Medical Center, 2200 Pierce Avenue, Preston Research Building, Nashville, TN 37232 USA; 7 University of Toulouse, Bat L3, I2MC/INSERM U1048, Avenue Jean Poulhès, 31432 Toulouse Cedex 4, France; 8 Department of Chemistry, Bridge Institute, Los Angeles, CA USA; 9 Heptares Therapeutics, Biopark, Welwyn Garden City, Hertfordshire AL7 3AX UK; 10 Pfizer Worldwide Medicinal Chemistry, Pfizer Neusentis, The Portway Building, Granta Park, Cambridge, CB21 6GS UK; 11 Membrane Protein Laboratory at Diamond Light Source, Didcot, UK; 12 Molecular Therapeutics, School of Pharmacy, Medical Biology Centre, Queen’s University Belfast, 97 Lisburn Road, Belfast, BT9 7BL Northern Ireland UK; 13 Boehringer Ingelheim Pharma GmbH & Co KG, Biberach, Germany; 14 Optibrium Ltd, 7221 Cambridge Research Park, Beach Drive, Cambridge, CB25 9TL UK; 15 UCB Pharma, 216 Bath Road, Slough, SL1 3WE UK; 16 Hodgson Pharma Consulting Ltd, Stevenage Bioscience Catalyst, Stevenage, SG1 2FX UK; 17 Department of Biochemistry, University of Oxford, South Parks Road, Oxford, OX1 3QU UK

**Keywords:** GPCRs, G-protein coupled receptors, β_2_AR, β_2_-adrenergic receptor, GLP-1, Glucagon-like peptide-1 receptor, CCK_2_R, cholecystokinin receptor-2, δ-OR, delta-opioid receptor, CRF1, corticotropin releasing factor receptor 1, CXCR_1_, CXCR_2_, CCR_4_ and CCR_5_, chemokine receptors, 5-HT_2B_ and 5-HT_2C_, human 5-hydroxytryptamine receptors 2B and 2C, respectively, H_1_, histamine receptor 1, hM_3_R, human muscarinic M3 receptor, Dopamine D_2_ receptor, α1B Adrenergic receptor, T4L, T4-lysozyme, BRIL, apocytochrome b_562_RIL, XFELs, x-ray free electron lasers, SDM, site-directed mutagenesis, MD, molecular dynamic simulations, 3D, three-dimensional, 7TM, seven-transmembrane domain, TM, trans-membrane helix, ECL, extracellular loop, HGMP, hierarchical GPCR modelling protocol, GLAS, GPCR-likeness assessment score, ProS, pairwise protein similarity method, PDB, Protein Data Bank

## Abstract

G-protein coupled receptors (GPCRs) are the targets of over half of all prescribed drugs today. The UniProt database has records for about 800 proteins classified as GPCRs, but drugs have only been developed against 50 of these. Thus, there is huge potential in terms of the number of targets for new therapies to be designed. Several breakthroughs in GPCRs biased pharmacology, structural biology, modelling and scoring have resulted in a resurgence of interest in GPCRs as drug targets. Therefore, an international conference, sponsored by the Royal Society, with world-renowned researchers from industry and academia was recently held to discuss recent progress and highlight key areas of future research needed to accelerate GPCR drug discovery. Several key points emerged. Firstly, structures for all three major classes of GPCRs have now been solved and there is increasing coverage across the GPCR phylogenetic tree. This is likely to be substantially enhanced with data from x-ray free electron sources as they move beyond proof of concept. Secondly, the concept of biased signalling or functional selectivity is likely to be prevalent in many GPCRs, and this presents exciting new opportunities for selectivity and the control of side effects, especially when combined with increasing data regarding allosteric modulation. Thirdly, there will almost certainly be some GPCRs that will remain difficult targets because they exhibit complex ligand dependencies and have many metastable states rendering them difficult to resolve by crystallographic methods. Subtle effects within the packing of the transmembrane helices are likely to mask and contribute to this aspect, which may play a role in species dependent behaviour. This is particularly important because it has ramifications for how we interpret pre-clinical data. In summary, collaborative efforts between industry and academia have delivered significant progress in terms of structure and understanding of GPCRs and will be essential for resolving problems associated with the more difficult targets in the future.

## Introduction

**Fig. 1:**
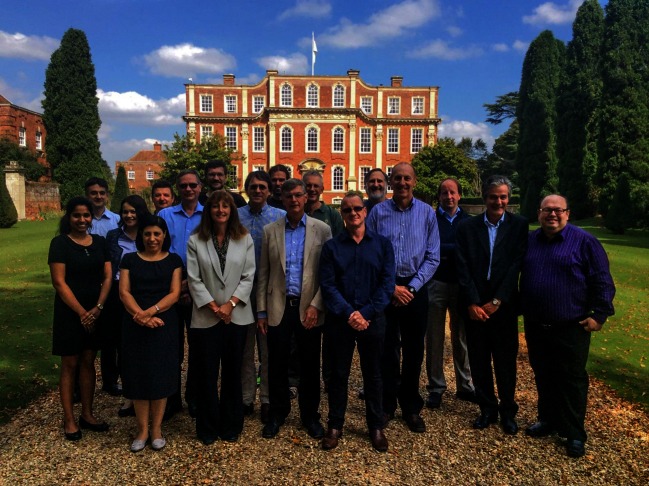
Attendees of the conference (listed from left to right): front row—Isabel Moraes, Fiona Marshall, Gebhard Schertler and Patrick Sexton; second row—Shweta Singh, Irina Tikhonova, Tom Ceska, Roland Seifert, Simon Hodgson, Daniel Fourmy and Alexander Heifetz; back row—Ian Storer, Mike Bodkin, Vadim Cherezov, Christofer Tautermann, Christopher Tate, Vsevolod Gurevich and Peter Hunt. Chicheley Hall itself can be seen in the background (the photograph was taken by Richard Law)

The Royal Society Academia-Industry International Conference 2014 focussed on the topic of ‘GPCR Structure, Function, Drug Discovery and Crystallography’ and was held on September 1–2 in Chicheley Hall, UK. This conference brought together 20 renowned experts in GPCR research and drug discovery spanning Europe, Australia and North America. Approximately half of the attendees were from academia and half from industry (see Fig. 1).

GPCRs are a large family of integral membrane proteins that have enormous physiological and biomedical importance. Since GPCRs are involved in mediating cell signalling processes, they are implicated in many diseases and are the targets of numerous therapeutic drugs. This renders GPCRs one of the most important classes of current pharmacological targets. This is borne out by the fact that 60 % of all prescription drugs today target GPCRs (Schöneberg et al. 2004), developed for just 50 established GPCR targets out of the 800 known members of the gene family. The importance of GPCRs was recently highlighted with the Nobel Prize for Chemistry 2012 being awarded to two eminent GPCR researchers, Prof. Brian Kobilka (Stanford University, CA, USA) and Prof. Robert Lefkowitz (Duke University, NC, USA). There remains an ongoing need to better understand the interplay between structure and function of these receptors to advance our scientific knowledge and capacity to more effectively harness therapeutic capabilities. As a result, GPCR crystallography and modelling are rapidly expanding. However, there is still a sizeable gap between ongoing academic research and the needs of the pharmaceutical industry. A major reason for this is that academic and industrial scientists have too few productive opportunities to meet and interact, particularly to establish cross-discipline collaborations. The intimate atmosphere of such a small conference provided a unique opportunity to stimulate the generation of new networks and partnerships between academia and industry, and to promote current GPCR research and its applications to drug discovery. The invitation of scientists representing structural biology, protein engineering, pharmacology as well as computational and medicinal chemistry provided an interdisciplinary core to enable fruitful discussion and debate.

Many of the presentations stressed the importance of collaboration. For example, Prof. Schertler pointed out that during 18 years at the MRC Lab in Cambridge, he had a continuous string of collaborations with industry partners from small and large Pharma that created a very valuable network of complementary expertise. This collaborative network led to breakthroughs in the expression and purification of difficult membrane protein targets, and later this network was an important ingredient in the formation of the drug discovery spin out company Heptares Therapeutics. Several of Prof. Schertler’s earlier industry partners became the drivers of this GPCR-oriented company with their expertise on target selection, business models and intellectual property (IP) complemented perfectly the expertise of the MRC academic partners. The spin out company has in the meantime grown from three post docs to about 80 people, and it is able to tackle the most difficult GPCR targets with resources that would otherwise not have been accessible in Europe from any funding agency.

The basis of good industry academia collaboration is a clear agreement about the goals of the collaboration. Very often selecting a pre-competitive goal allows the academic partner to fully publish the results and gives the industry partner a significant edge in accessing emerging technologies. The example of Heptares Therapeutics illustrates that a spin out company can generate resources that would neither be available inside a company or from public funding agencies. If the spin out is able to reach milestones and refinance, then it can become a powerful tool to drive the application of new technology and lead to a change in the research culture in industry.

Most ventures between academia and industry are dependent on track record and trust. Individuals have to commit to a longer-term perspective, which is aimed at changing the scientific landscape. This scientific environment is essential for large companies that exist and are established to be able to recruit an excellent workforce and for small companies to pick up competitive innovation. In multinational companies, the academic and technology environment can start to dictate the location of departments inside a large organisation. From this follows that for a stable business development, the academic environment is as essential to the company as the in-house research activities.

One of the most significant advances in previous years has been the structural advances, both in terms of stabilising protein conformational states to make them more amenable to x-ray crystallography, but also in new technology such as x-ray free electron lasers which have the potential to accelerate structural biology not just for GPCRs but for many integral membrane protein drug targets. A significant portion of the meeting discussed these advances and also how they had been used in recent drug-discovery programs within both industry and academia. Another focus in recent times has been the concept of ‘ligand bias’. It has become clear that individual GPCRs can exist in multiple receptor conformations and can elicit numerous functional responses, both G protein- and non-G protein-mediated. This has led to the discovery that different ligands can stabilise distinct subsets of receptor conformations that can ‘traffic’ stimulus to diverse functional outputs with varying prominence, a concept referred to as biased signalling (also known as functional selectivity, stimulus bias or ligand-directed signalling). In principle, biased signalling can result in the development of more efficacious and safer drugs, but there are some unresolved questions regarding the best system in which to assess these aspects. The structural information alongside the realisation of biased signalling has also been explored with in silico modelling, and it was demonstrated at the meeting that this can give very useful insight into underlying properties of signalling control. The final section of the meeting focussed on how to best resolve problems in the future, including modelling processes at a higher level. In the following sections, we expand on these discussions in more detail.

## Developments in GPCR crystallography

**Fig. 2:**
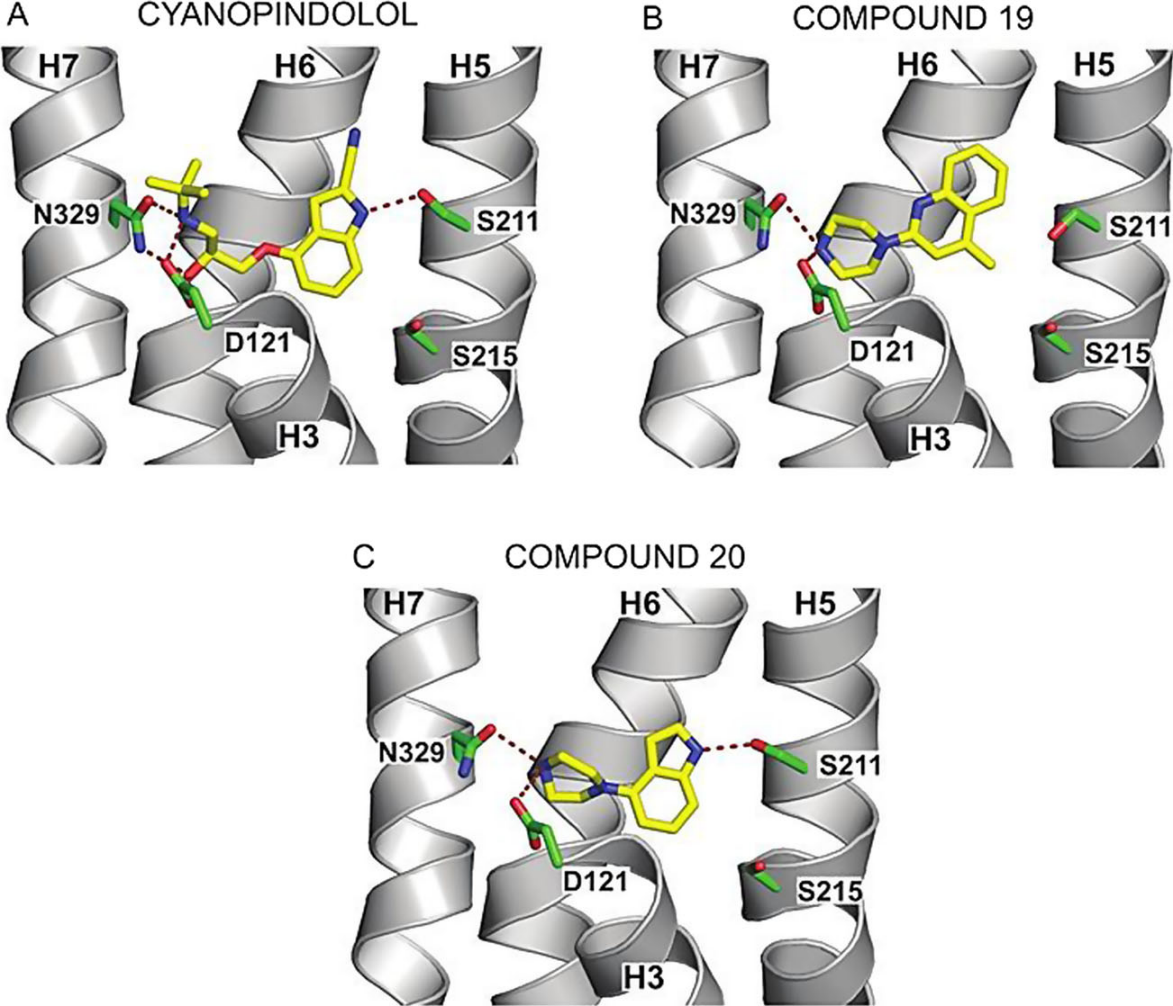
Crystal structures of β_1_AR bound to novel chemotypes developed by fragment screening and hit optimisation. Structures of the ligand binding pocket are depicted with only portions of selected transmembrane helices (H3, H5, H6 and H7) shown with the side chains (*green*) and ligands (*yellow*) depicted in sticks: **a** cyanopindolol, PDB code 2VT4; **b** compound 19, PDB code 3ZPQ; **c** compound 20, PDB code 3ZPR—figure adapted from the publication (Christopher et al. 2013)

It is apparent from numerous studies that the stability of the GPCR-ligand complex in detergent solution is an important parameter that will dictate the success of any crystallisation trials (Tate 2012). Although high thermostability alone does not guarantee the formation of diffraction-quality crystals, if the GPCR-ligand complex is too unstable, then crystals may not form or, if they do, they may diffract only poorly. The majority of GPCR structures have been determined from crystals of the receptor bound to a high-affinity antagonist, which usually binds with *K*_d_ or *K*_i_ values in the range of 10 pM to 10 nM. However, if a ligand binds to a receptor only with low affinity and/or the receptor is unstable in detergent, then it may still be possible to obtain a structure if the receptor is thermostabilised. A method developed to thermostabilise GPCRs uses systematic scanning mutagenesis coupled to a thermostability assay performed on the detergent-solubilised mutant receptors to identify specific thermostabilising mutations (Tate 2012). Each mutation usually imparts 1–3 °C improvement in thermostability to the receptor, although the most highly stabilising mutation found improved thermostability of the agonist-bound conformation of the adenosine A_2A_ receptor by 14 °C. Once the single thermostabilising mutations have been identified, then the best thermostabilising mutations can be combined to make an optimally stable receptor (Shibata et al. 2013). The methodology has been applied to many different GPCRs, in either an antagonist-bound conformation or an agonist-bound conformation, which have been subsequently crystallised and their structures determined. The most recent structure (Miller-Gallacher et al. 2014) was a 2.1-Å resolution structure of the β_1_-adrenergic receptor (β_1_AR) bound to cyanopindolol (see Fig. 2) and the crystals grown in lipidic cubic phase, although without requiring fusions of the receptor to either T4-lysozyme (T4L) or apocytochrome b_562_RIL (BRIL). The structure showed the presence of an intramembrane sodium ion that was in the identical position to the intramembrane sodium ion in the adenosine A_2A_ receptor that is known to act as an allosteric antagonist. In contrast, the Na^+^ ion in β_1_AR does not appear to affect the transition between the inactive and active states of the receptor.

A significant advantage of the thermostabilised receptors is their use in drug discovery, which for the first time opens up the opportunities for structure-based drug design (Congreve et al. 2014). The thermostabilised receptors are readily purified and crystallised on a routine basis, which facilitates co-crystallisation with fragments and lead compounds. This was recently demonstrated for the β_1_AR where a fragment screen performed by surface plasmon resonance (SPR), followed by minimal hit optimisation, produced nM-affinity antagonists with novel scaffolds that were readily co-crystallised with the receptor (Christopher et al. 2013). The thermostabilisation of GPCRs is a central platform in Heptares Therapeutics, resulting in numerous crystal structures.

Another structural approach gaining momentum is the application of x-ray free-electron lasers (XFEL) to GPCRs. Structural studies of GPCRs, and other biomedically relevant membrane proteins and complexes, are hampered by challenges related to growing sufficiently large crystals capable of withstanding radiation damage and yielding high-resolution data at synchrotron sources. The recent introduction of a new generation of x-ray sources, x-ray free electron lasers (XFELs), producing ultra-bright pulses of coherent x-rays with an ultrashort duration, holds the promise to advance our understanding of structure and function of these challenging targets.

**Fig. 3:**
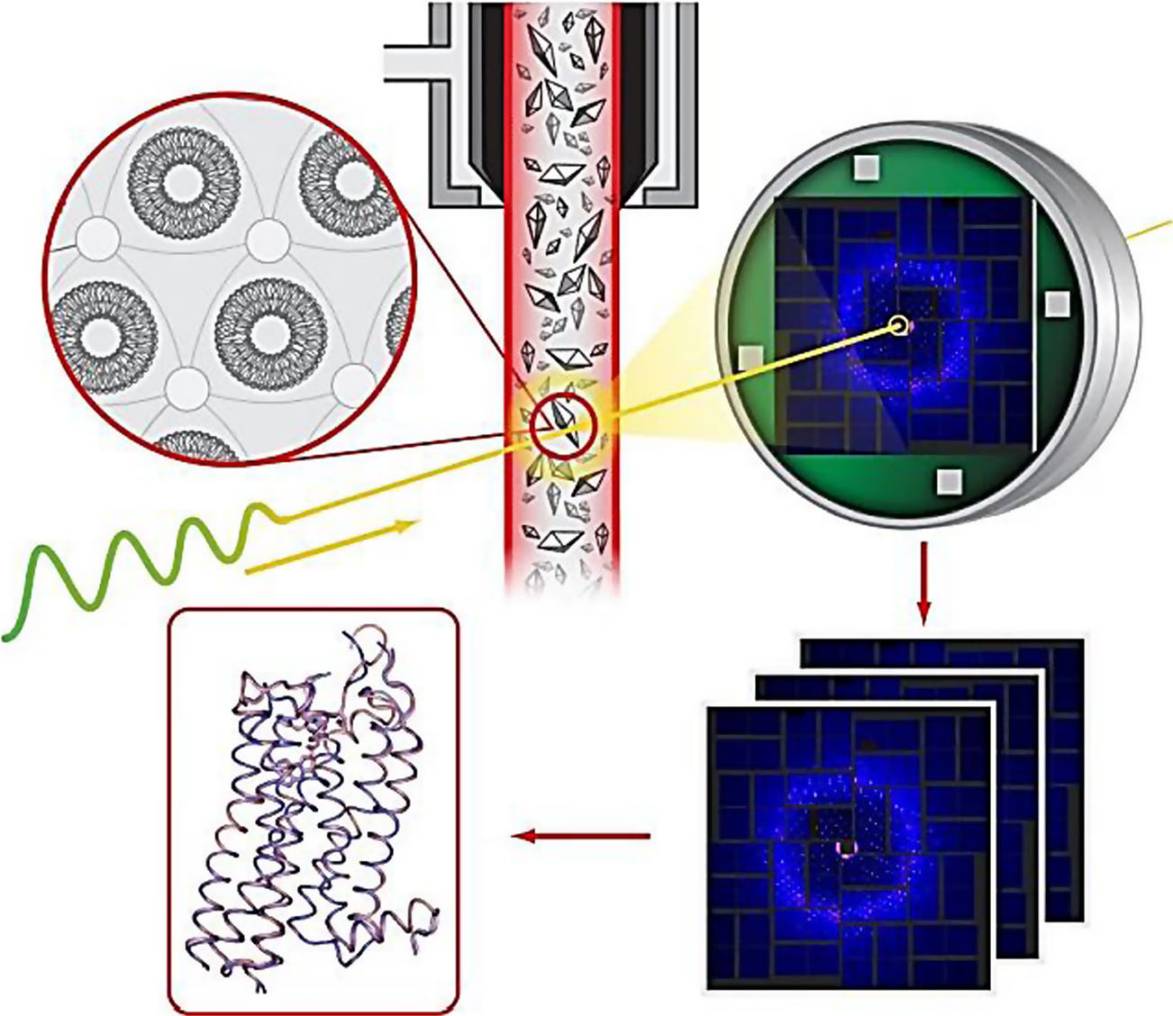
Schematic summary illustrating serial femtosecond crystallography of GPCRs with using lipidic cubic phase for microcrystal growth and delivery

A novel approach using a membrane mimetic gel-like matrix known as lipidic cubic phase (LCP) for growth and delivery of membrane protein microcrystals for data collection by serial femtosecond crystallography (SFX) at XFELs (Liu et al. 2013) was described. Microcrystals are delivered to the intersection point with an XFEL beam in random orientations using a specially designed LCP injector (Weierstall et al. 2014). The injector allows adjusting LCP flowrate in a wide range to match the XFEL pulse repetition rate and, thus, minimises crystal consumption. LCP-SFX uses highly intense sub-50-fs XFEL pulses to overcome radiation damage and collect room temperature high-resolution data from sub-10-μm crystals. Protein consumption is reduced by two to three orders of magnitude compared to previously used liquid injectors, making the LCP-SFX method attractive for structural studies of challenging membrane and soluble proteins, and their complexes (see Fig. 3) (Liu et al. 2014a, Liu et al. 2014b).

Results demonstrating the great utility of this approach were highlighted at the meeting. They included the structure of the human delta-opioid receptor bound to a bi-functional peptide ligand (Fenalti et al. 2014), the structure of a major GPCR signalling complex (unpublished data) and the first novel GPCR structure solved entirely by the LCP-SFX approach (unpublished data). In the future, this method could lead to the development of an efficient GPCR structure-based drug design pipeline by removing the major obstacles, such as the difficulties in preparation of large amounts of homogeneous and stable protein and growing sufficiently large crystals for a large number of different protein-ligand complexes. Another advantage of XFELs is the ability to freeze protein motion in time and obtain structures of unstable intermediates, advancing our knowledge about the signal transduction mechanism in GPCRs.

It was also noted that there are many signalling complexes in the cell that have component parts that have some flexibility as part of the structure necessary for function. Where these structures are difficult to stabilise, crystallisation is often problematic. Quite often crystallography can stumble because only very small crystals are attainable, with very weak diffraction, and this can be due, in part, to some partial disorder in a biologically relevant part of the molecule. The crystallographic solution up to now has been to delete or modify the flexible regions of the protein in order to create constructs that are more ordered so that crystals of significant size could be grown. With diffraction from microcrystals now possible, the structures of these more challenging biological assemblies are within reach.

## Improvements in the understanding of GPCR function

In terms of function, the human β_2_-adrenergic receptor (β_2_AR) is probably the best-studied GPCR at the molecular, cellular and physiological level (Seifert 2013). The β_2_AR was of critical importance for the development of current models of receptor activation including biased signalling. Recent research has also shown for this system that ligand bias can depend on the system studied (native versus recombinant) (Seifert 2013) and also on the (patho)physiological state (healthy versus diseased), a feature sometimes also referred to as dynamic bias (Michel et al. 2014). Accordingly, the analysis of ligands at receptors such as the β_2_AR has become much more complex, requiring multidimensional approaches.

The β_2_AR constitutes an important drug target; agonists for this receptor are used for treatment of bronchial asthma and chronic-obstructive lung disease. However, safety and efficacy of β_2_AR agonists are not optimal. Most strikingly, the use of β_2_AR agonists alone in asthma is associated with increased mortality. Receptor desensitisation and activation of deleterious non-canonical signalling pathways, i.e. pathways different from the canonical G_s_ pathway, could contribute to this situation. Moreover, specific β_2_AR polymorphisms may be associated with decreased responsiveness to certain ligands, and the use of racemic β_2_AR ligands may be problematic. Specifically, the distomeric (not therapeutically active) ligands may contribute to drug toxicity (Seifert and Dove 2009).

**Fig. 4:**
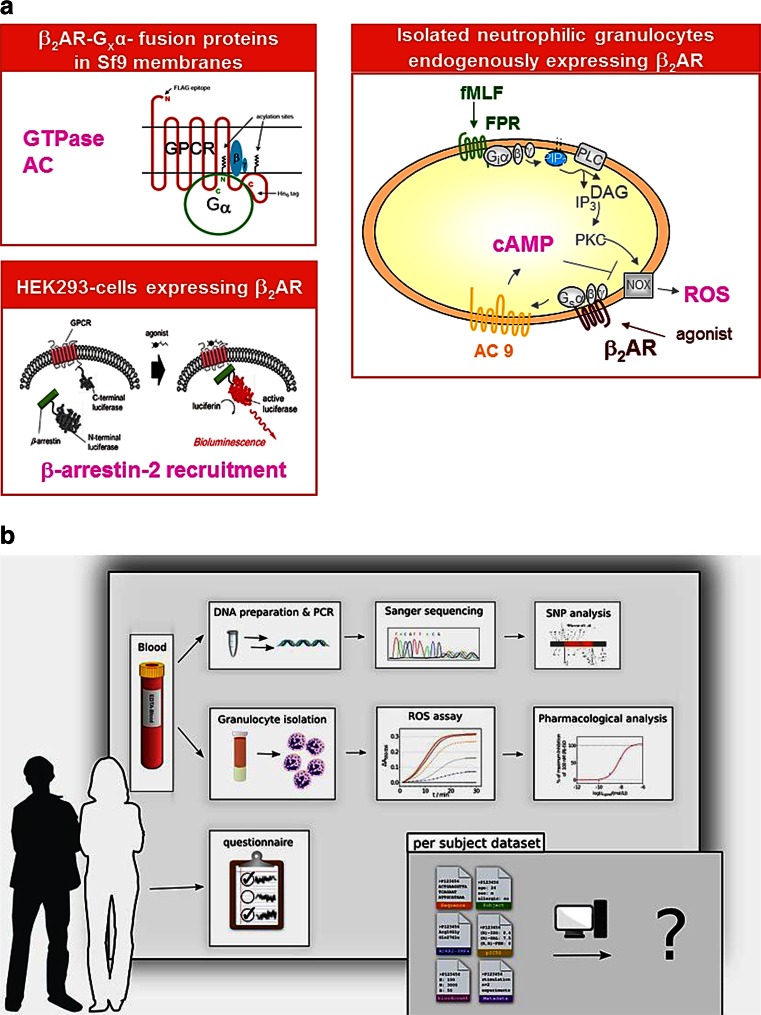
**a** Multidimensional analysis of the β_2_AR in native and recombinant systems. Study design. The concept of functional selectivity requires that a given GPCR is analysed in multiple different assays. GPCR-Ga fusion proteins ensure a defined 1:1 stoichiometry of receptor and G-protein and allow analysis of GDP/GTP turnover, measured in the GTPase activity assay, and effector system activation, measured in the adenylyl cyclase (*AC*) assay, with high sensitivity. The measurement of β-arrestin recruitment is accomplished in HEK cells stably transfected with fusion proteins of the β_2_AR linked to a luciferase fragment and β-arrestin linked to the complementary luciferase fragment. Upon binding of an agonist, β-arrestin binds to the GPCR, and luciferase activity is reconstituted. Human neutrophilic granulocytes constitute a physiologically relevant model system for the β_2_AR. In these cells, the β_2_AR couples to AC (isoform 9), resulting in an increase in the second messenger cAMP. The β_2_AR inhibits formyl peptide receptor (*FPR*)-mediated activation of the neutrophilic NADPH oxidase (*NOX*) that generates reactive oxygen species (*ROS*). FPR-mediated NOX activation is inhibited by the β_2_AR. It is generally assumed that this inhibition is mediated via cAMP, but an increasing number of studies indicate that the inhibition is actually cAMP independent. **b** Functional and genetic analysis of β_2_AR polymorphisms in 60 healthy volunteers. Study design. A major goal of current pharmacological research is the development of individualised pharmacotherapy that takes into account individual genetic polymorphisms. At the level of GPCRs, very little research has been performed in this field so far. Therefore, as a model receptor, the β_2_AR was analysed because for this GPCR several polymorphisms are already known, but assignment of specific polymorphisms to defined disease entities is controversial. After obtaining consent from volunteers and completing a questionnaire, a small sample of blood (4–8 ml) was drawn from healthy male and female subjects. A fraction of the blood was used to sequence the β_2_AR gene to identify known (and unknown) β_2_AR polymorphisms. The remainder of the blood was used to isolate human neutrophils and assess the pharmacological profile of the β_2_AR with several standard ligands according to the signalling paradigm shown in (**a**). Ligands were characterised with regard to potency and efficacy. The data sets have now been completed. The pharmacological profile of each individual was assessed several times. Currently, data are analysed in multiple ways. Specifically, the impact of specific β_2_AR polymorphisms on the pharmacological profile is assessed. Additionally, the impact of sex, age, smoking and allergy history on β_2_AR pharmacology is evaluated. The study fills a gap in the field because it provides data on the pharmacological properties of GPCR polymorphisms in a physiologically relevant context. The results of the study will be submitted for publication to a peer-reviewed journal in spring 2015

Based on these concerns, non-canonical signalling pathways, receptor polymorphisms and pure β_2_AR stereoisomers were examined in a pluridimensional signal transduction matrix. This matrix included studies with recombinant β_2_AR and native β_2_AR expressed in human neutrophils (see Fig. 4). Human neutrophils constitute a relevant cell type for inflammation in bronchial asthma that can be readily isolated in substantial numbers. In neutrophils, the β_2_AR exerts anti-inflammatory effects by inhibiting chemoattractant-stimulated superoxide radical formation. Steroisomers of fenoterol were used as model ligands because these ligands have already been shown to exhibit functional selectivity (Seifert and Dove 2009).

In aggregate, these studies revealed that most reported ligands are biased towards canonical G_s_ signalling (Reinartz et al. 2015). This is particularly evident for ligands with large *N*-alkyl substituents, suggesting that these ligand domains, through constrained mobility of transmembrane helices, impede with coupling to G_i_ proteins and β-arrestin. In principle, this pharmacological pattern of fenoterol stereoisomers should be favourable for asthma treatment. However, the Seifert group did not identify any ligand with bias towards G_i_ or β-arrestin.

With regard to polymorphisms, 60 healthy volunteers were studied (see Fig. 4b). From these volunteers, blood was drawn and neutrophils were isolated. The pharmacological profile of the β_2_AR with respect to inhibition of superoxide radical formation was assessed. Moreover, the β_2_AR gene of the individuals was sequenced. Overall, substantial variability in the pharmacological profile of the β_2_AR in neutrophils was noted, but no association of the pharmacological β_2_AR profile with a specific polymorphism emerged. Thus, at the time being, there is no evidence for the notion that β_2_AR polymorphisms can be used to optimise asthma therapy.

To summarise this aspect, our increase in knowledge of the β_2_AR has resulted in a situation that renders future research more complicated. Most importantly, it is not anymore sufficient to determine a single parameter for a receptor such as G_s_-mediated adenylyl cyclase activation. Rather, multiple parameters have to be determined including non-canonical G_i_- and β-arrestin signalling. It is important to analyse the β_2_AR not only in recombinant but also in native systems. It will also be very informative to resolve crystal structures of the β_2_AR in complex with various ligands and coupling partners to understand the molecular basis of functional selectivity (Seifert and Dove 2009).

The GLP-1 receptor represents a good model system for studying class B receptor function (Koole et al. 2013a, b; Wootten et al. 2013a). GLP-1 is a key incretin peptide that promotes insulin secretion in response to nutrient ingestion, but also has a range of other actions including preservation of B-cell mass, reduction in gastric emptying and reduction in appetite that make it a desirable target for treatment of type II diabetes. Class B secretin-like receptors, like many other GPCRs, are pleiotropically coupled to a spectrum of both G-protein-dependent and -independent signalling pathways, and while cAMP production is the best characterised signalling endpoint for these receptors, physiological and therapeutic responses are the product of the integrated signalling response from all activated pathways. Moreover, the different contacts that are made between distinct ligands and their respective receptor can engender unique receptor conformations that give rise to distinct signalling profiles. This behaviour can be observed through differences in activation of second messengers, but also through changes to how receptors are desensitised and down-regulated. Biased signalling is further complicated when allosteric drugs are considered, as conformational preferences of the receptor when allosteric and orthosteric (endogenous ligand) sites are co-occupied may be different than when either site is individually occupied. Biased signalling is particularly relevant to receptor systems that have multiple endogenous ligands, and where exogenous mimetics are used clinically, as is often seen with class B receptors.

Clear (ligand-directed) bias for both peptides and small molecule agonists of the GLP-1 was demonstrated (Koole et al. 2013a, b), and this is consistent with earlier studies of pituitary adenylyl cyclase-activating polypeptide type 1 receptor (PACAPR) receptors and unclassified class B members like hCTRs, suggesting that this is likely to be a common feature of agonist activation of class B receptors. Transmembrane helical packing and conformational transition involved in receptor activation are assumed to involve key hydrogen bond networks formed around polar residues in the transmembrane helices.

The importance of conserved polar residues in class B receptors at the GLP-1 receptor has been recently studied (Koole et al. 2013a; Wootten et al. 2013b). This work has revealed networks of interaction that differentially contribute to global receptor activation and biased signalling. In particular, there appear to be two key interaction networks, one at the base of the receptor that may serve a similar role to the D[E]RY motif in class A receptors to maintain an inactive state; the second is located in the mid-region of the helical core and plays a critical role in pathway specific signalling, in a ligand-dependent manner. Mutation of residues within the central network has identified differences in how ligands propagate activation transition for individual signalling pathways and that distinct ligands utilise only subsets of the network for signal propagation providing initial insight into molecular mechanisms for biased signalling.

**Fig. 5:**
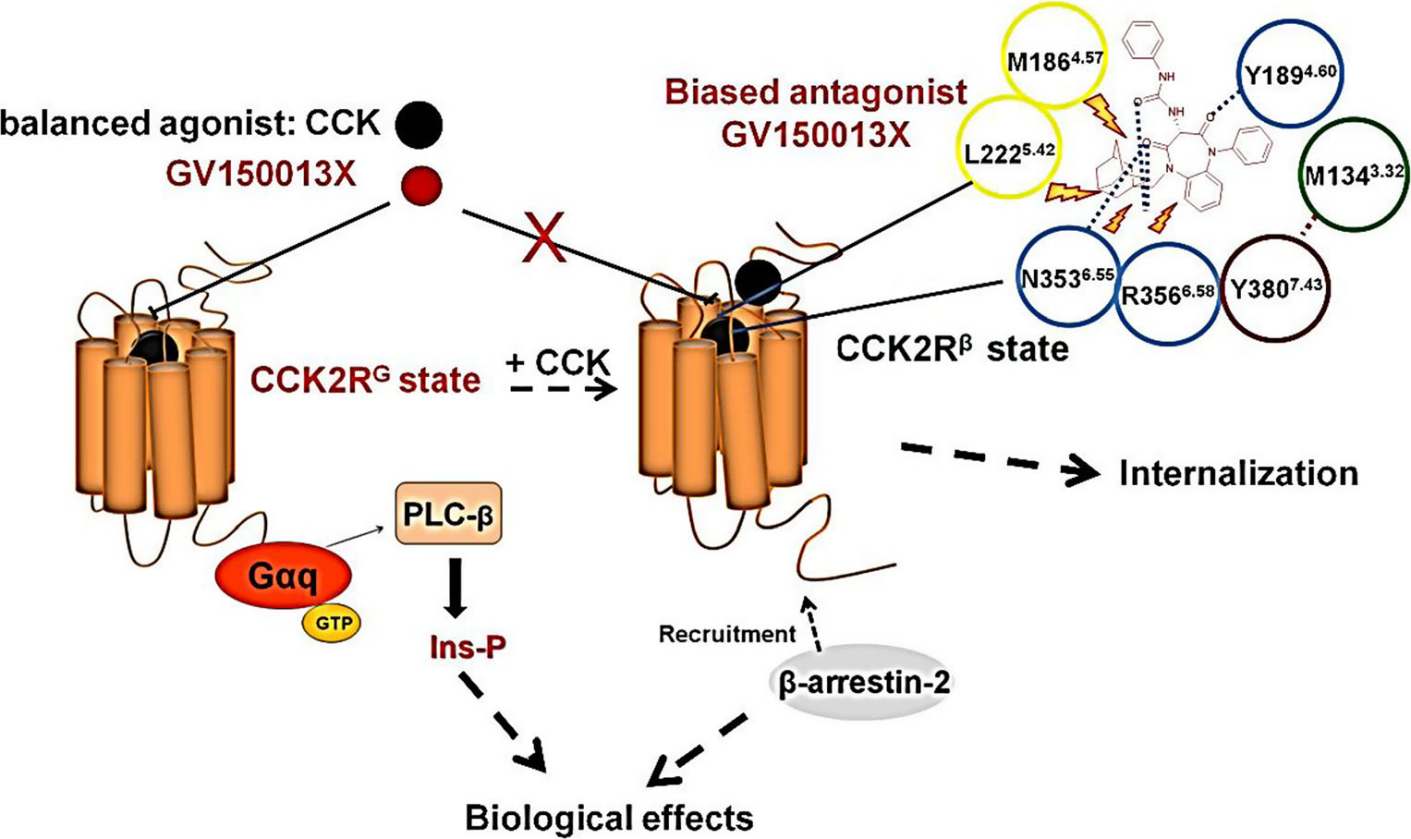
Schematic representation of the CCK_2_R which can adopt two distinct conformational states upon CCK activation. The CCK_2_R^G^ state couples to phospholipase-C activation and the CCK_2_R^β^ state recruits β-arrestins. This figure also shows that GV150013X, a competitive antagonist on CCK_2_R^G^, is inefficient to inhibit recruitment of β-arrestins by CCK_2_R^β^ because of steric hindrance at the orthosteric binding site

The concept of biased signalling has also been explored in the cholecystokinin receptor-2 (CCK_2_R, which also binds the digestive hormone gastrin) (Magnan et al. 2011, 2013) and is a GPCR for which pharmaceutical companies and academic laboratories have successfully developed non-peptide ligands, mostly antagonists. Since CCK_2_R is a potential target in several pathologies of the central nervous system (anxiety, panic attacks), of the gut (peptic ulcer disease) and of neuroendocrine cancers, the effects of a series of such ligands on stimulation of phospholipase-C and as well as on recruitment of non-visual arrestins and stimulation of receptor internalisation have been studied (see Fig. 5).

Several ligands stimulating phospholipase-C with efficacies reaching up to 50 % of that of CCK but which were inefficient to stimulate β-arrestin1/2 recruitment and receptor internalisation have been identified (Magnan et al. 2011). These ligands, which were initially considered as full antagonists at the CCK_2_R, are therefore more accurately described as antagonists on the β-arrestin-dependent signalling pathway of this receptor, but are partial agonists of the G-protein-dependent signalling pathway.

Extending these studies to ligands that are antagonists of G-protein-dependent pathways, it was discovered that the antagonist termed GV150013X (*N*-(+)-[1-(Adamant-1-ylmethyl)-2,4-dioxo-5-phenyl-2,3,4,5-tetrahydro-1*H*-1,5-benzodiazepin-3-yl]-*N*′-phenylurea) (see Fig. 5) could not inhibit β-arrestin1/2 recruitment and CCK-induced CCK_2_R internalisation (Magnan et al. 2013). Schild plot analysis of antagonist activity of GV150013X on CCK-induced phospholipase-C activation indicated that this molecule competitively inhibited the CCK effect. This information led the team to dock GV150013X in the orthosteric binding site of the modelled CCK_2_R, which had been previously validated by site-directed mutagenesis. The docking study predicted that the absence of effect of GV150013X on CCK_2_R recruiting β-arrestins was due to the presence of a bulky adamantane moiety in the ligand. GV150013X competitively inhibited CCK-induced G-protein-dependent pathway whereas it was inefficient on the β-arrestin-dependent pathway. These data suggested that the CCK_2_R could adopt two distinct conformations upon CCK activation and these two conformations are distinguishable at the binding site level. Fourmy’s lab therefore performed a site-directed mutagenesis study of the CCK_2_R orthosteric binding site with a focus on amino acids presumably in contact with the adamantane moiety of GV150013X. In parallel, the team synthesised an analogue of GV150013X in which the adamantane moiety was substituted by a methyl group: 1-(2,3,4,5-tetrahydro-1-methyl-2,4-dioxo-5-phenyl-1H-benzo[*b*][1,4]diazepin-3-yl)-3-phenylurea, termed GV-CH3. Pharmacological studies with CCK_2_R mutants and with GV-CH3 consistently demonstrated that the prediction of modelling and docking study was correct: the absence of effect of GV150013X on recruitment of β-arrestins to the CCK_2_R was due to a steric hindrance within the binding site which impedes binding of the antagonist (Magnan et al. 2013).

Overall, this area of research shows how pharmacological analysis of GPCR signalling combined with molecular modelling of GPCRs and chemistry of ligands can be used to analyse the origin of biased signalling. Such a strategy together with forthcoming determination of GPCR structures in complex with various signalling proteins (G proteins, β-arrestins) opens the possibility of rational drug design of biased ligands.

One of the biased-agonism pathways involves arrestins, and they themselves present an alternative line of investigation. Arrestins specifically bind active phosphorylated GPCRs, precluding further G protein activation and channelling the signalling to G-protein-independent pathways (Gurevich and Gurevich 2006). Based on the elucidation of an arrestin structure and key functional elements, special arrestins to channel cell signalling in a desired direction were constructed (Gurevich and Gurevich 2012). Enhanced phosphorylation-independent arrestin mutants were designed by disrupting key stabilising intra-molecular interactions that hold arrestins in a basal conformation (Gurevich and Gurevich 2012). Enhanced arrestin-1 was shown to compensate for the defects of rhodopsin phosphorylation in vivo, prolonging the survival of mutant rod photoreceptors, improving their light sensitivity and speeding up photoresponse recovery (Song et al. 2009). While the compensation with the first-generation enhanced mutant was only partial, new more powerful phosphorylation-independent forms of arrestin-1 hold promise for a better compensation (Vishnivetskiy et al. 2013). In rod photoreceptors, rhodopsin-specific arrestin-1 is the prevalent form, so it is clear that one needs to target this subtype to compensate for disease-causing defects of rhodopsin phosphorylation.

However, activating mutations in many GPCRs underlie various human disorders (Schöneberg et al. 2004). Since the two non-visual arrestins are fairly promiscuous, interacting with hundreds of GPCR subtypes, and most cells express 5–25 different GPCRs, only one of which is a mutant, to use a compensational approach, one needs receptor-specific non-visual arrestins. To this end, the elements of non-visual arrestins that determine their receptor preference were identified (Vishnivetskiy et al. 2011), and on the backbone of the most promiscuous non-visual subtype, arrestin-3, mutants with high (>50-fold) receptor specificity were created (Gimenez et al. 2012). This finding showed that targeting individual receptors with engineered non-visual arrestins is feasible. Arrestins interact with numerous partners, organising multi-protein complexes and recruiting them to particular sub-cellular compartments (Gurevich and Gurevich 2014a, b). This creates the potential of constructing signalling-biased arrestins that activate or inhibit certain pathways without affecting others. Recently, Gurevich and colleagues designed an arrestin-3 mutant that acts as a silent scaffold: it binds all kinases in the c-Jun N-terminal kinase (JNK) activation cascade, but does not promote its phosphorylation (Breitman et al. 2012). This mutant was shown to act in a dominant-negative fashion, suppressing JNK activation in the cell (Breitman et al. 2012). Multi-functionality of arrestins suggests that parts acting on particular pathways can be separated and used to modify cell signalling. Indeed, a small element of arrestin-3 that acts as a mini-scaffold, promoting JNK activation in vitro and in cells, has been identified (Zhan et al. 2014). Anti-proliferative activity of this element can be used for therapeutic purposes. Since arrestins play a role in numerous signalling pathways, targeted mutagenesis can yield arrestin-based molecular tools to tell the cell what to do in a language it cannot disobey (Gurevich and Gurevich 2014a, b).

## Challenges and solutions for GPCR drug discovery

Despite many examples of successful marketed drugs that modulate the function of GPCRs, there remain a large number of potential therapeutically relevant GPCRs that are regarded as difficult to drug effectively. Methods used to analyse the GPCR’s ligand binding sites with a view to designing ligands were reviewed and included a discussion on the importance of water molecules for mediating interactions between ligands and receptor.

**Fig. 6:**
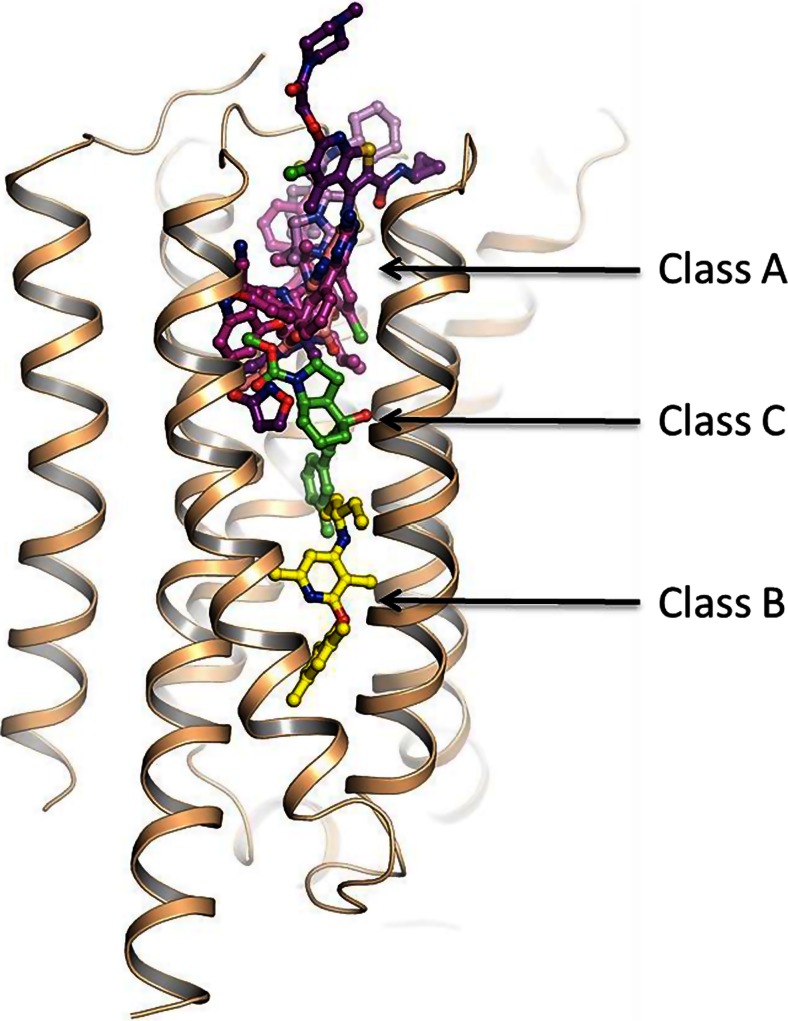
Crystal structure of the class C mGlu5-mavoglurant receptor (Bennett et al. 2014) complex with CP-376395 from the class B receptor CRF1 and overlays of a selection of ligands from class A receptor structures present in the PDB. The observed ligand binding positions demonstrate the spectrum of binding modes across GPCR classes ranging from extracellular orthosteric to deeper intracellular allosteric sites

Heptares use their StaR® technology to generate thermostabilised GPCRs which can be used for biophysical studies, fragment screening and determination of x-ray structures. Examples were presented from different GPCRs showing that the most potent ligands act to displace high energy or ‘unhappy’ waters deep within binding pockets. Water molecules can contribute to both ligand selectivity and kinetics. An overview of class B and class C structures recently solved at Heptares (see Fig. 6) was also presented. The corticotropin releasing factor receptor 1 (CRF1) x-ray structure identified a novel allosteric binding pocket deep within the transmembrane domain and illustrated why finding ligands for the orthosteric pocket has been challenging. The structure of class C metabotropic glutamate 5 receptor (mGlu5) was shown and provided an explanation for the tight structure-activity relationships (SARs) and pharmacology mode switching which have been observed for this receptor. The conclusions were that structure-based design could in theory now be applied more broadly across the GPCRome.

Antihistamines are one of the most well-studied drugs (Simons and Simons 2011). Indeed, it is now more than a century since the discovery of histamine as an important biogenic substance and more than 50 years since the production of the first antihistamine drugs (e.g. chlorpheniramine, diphenhydramine, hydroxyzine). H_1_R antagonists (antihistamines) are widely used in the treatment of a broad range of allergic diseases like rhinitis, conjunctivitis, urticaria and non-allergic disorders like pruritus and insomnia (Simons and Simons 2011). The first generation of H_1_R antagonists that had been introduced in the period of 1942–1980 demonstrated considerable side effects. Poor selectivity for the H_1_R and the ability to cross the blood–brain barrier (BBB) interfering with the histaminergic transmission (sedation) were among the most unwanted side effects. Recent studies have shown an increase in the number of allergic diseases, currently affecting more than 30 % of the world population (Qin 2007). Hence, there is an urgent need for more effective and safe anti-allergic drugs. The second-generation H_1_R antagonists, introduced in the early 1980s, had notable advantages such as being significantly more selective and non-sedating due to the lack of the ability to cross the BBB. These second-generation, non-sedating H_1_R antagonists have been widely used in the treatment of allergic conditions but still demonstrated some cardiotoxic side effects, e.g. induction of torsades-de-pointes arrhythmias. This has recently led to the development of third-generation H_1_R antagonists where both sedative and cardiovascular side effects were addressed (Oppenheimer and Casale 2002; Canonica and Blaiss 2011).

The crystal structure of the first-generation H_1_R antagonist doxepine bound to H_1_R was solved in 2011 (Shimamura et al. 2011). Doxepine has been associated with a large number of different side effects that can be rationalised by its lack of H_1_R selectivity and being a potent binder of H_2_R, some members of muscarinic, serotonic and α-adrenergic GPCR subfamilies and also of some protein kinases. Due to the lack of doxepine selectivity, the need for additional crystal structures of H_1_R bound to second and third generation of antihistamines that will rationalise the selectivity cannot be overestimated.

**Fig. 7:**
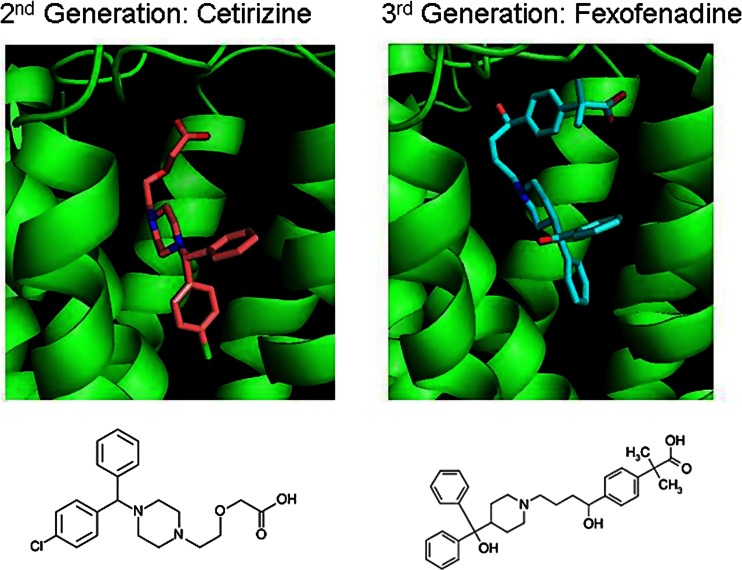
Crystal structure of H_1_R bound to highly selective second- and third-generation antihistamines: Cetirizine (*left*) and Fexofenadine (*right*)

Dr. Moraes reported for the first time the solution of two additional holo H_1_R crystal structures bound to the highly selective second- and third-generation H_1_R antagonists: Cetirizine (Gillard et al. 2002) and Fexofenadine (Sharma et al. 2014), respectively (unpublished data; see Fig. 7). Cetirizine and Fexofenadine are about 600-fold more selective for H_1_R compared with a wide panel of GPCRs and ion channels (Gillard et al. 2002). This work has resulted from collaboration between Evotec Ltd and the Membrane Protein Laboratory (MPL—Imperial College London).

These new structures provide insights into potency and selectivity, the key challenges for the design of new generation of H_1_R antagonists and provide a significant contribution to potentially aid computational guided structure-based drug discovery of new antihistamine drugs targeting H_2_, H_3_ and H_4_ receptors where crystal structures are still absent.

Structural information in conjunction with careful modelling can provide greater insight into the location and functional relevance of druggable binding locations including both orthosteric and allosteric sites. Several recent structural publications have provided greater clarity on the binding modes and kinetics of existing drugs in both orthosteric sites, such as bronchodilator tiotropium binding to muscarinic M_3_ receptor (Tautermann et al. 2013), and allosteric binding sites, such as anti-viral maraviroc which acts as a negative allosteric modulator of chemokine receptor CCR_5_ (Kruse et al. 2012; Tan et al. 2013). Provision of knowledge of this type should begin to assist in design of more subtype selective ligands, especially when combined with leading edge computational techniques such as homology modelling (Storer et al. 2014) and molecular dynamic simulations.

**Fig. 8:**
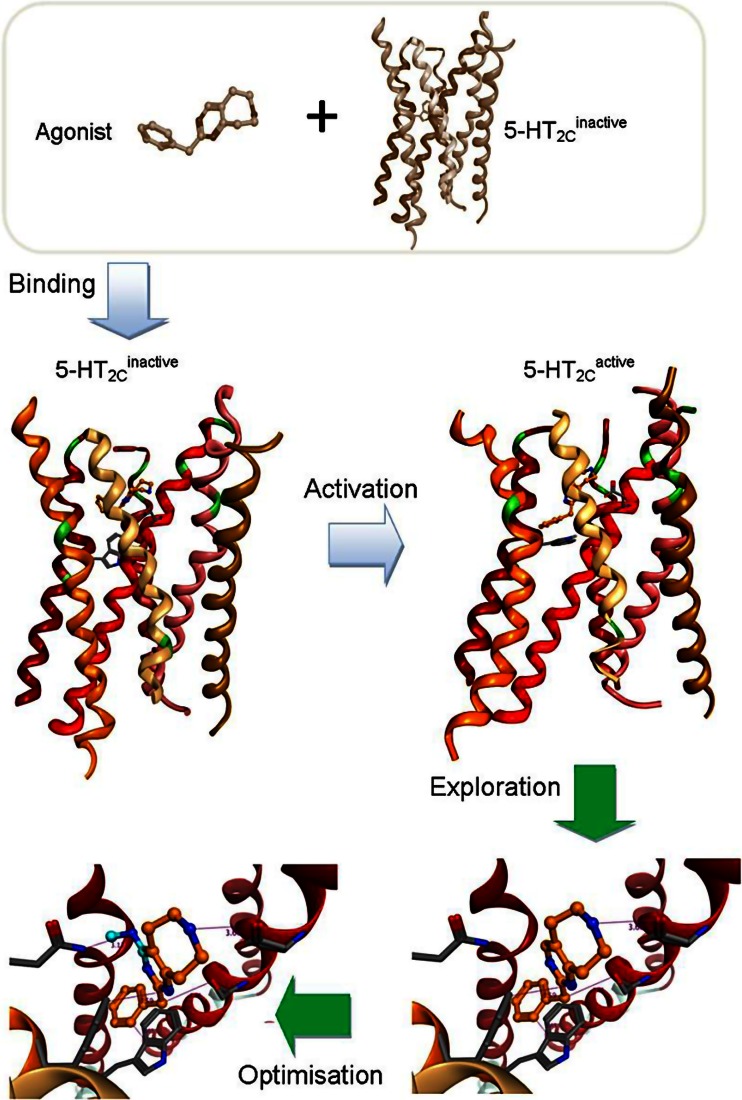
Modelling of binding and activation of 5-HT_2C_ (Storer et al. 2014) receptor by pyrimido[4,5-*d*]azepines

An area where increased structural knowledge could be especially impactful is in the improved design and optimisation of selective ligands. Selectivity between subfamily members of GPCRs has proved essential yet challenging in some instances, a notable example being the design of highly selective serotonin (5-HT) 5-HT_2C_-receptor agonists (see Fig. 8) (Monck and Kennett 2008).

Agonism of the 5-HT_2C_ receptor has therapeutic implications in a number of important disease areas including CNS disorders and obesity. However, the design of ligands that are selective over agonism of the closely related 5-HT_2B_ receptor, which has been linked with irreversible heart valvulopathy, has proved highly challenging (Storer et al. 2014). Furthermore, ligand-protein interaction features that lead to receptor agonism versus antagonism versus inverse agonism are not well understood despite numerous examples of subtle ligand structural changes driving pronounced differences in functional efficacy of GPCRs (Storer et al. 2014).

This is further complicated in instances where the same ligand exerts a different functional effect in different isoforms of the same receptor. This has been demonstrated by differential effects in the human isoform compared to the equivalent preclinical species receptors, hampering both in vivo efficacy and safety studies. An illustrative example is the histamine H_4_ receptor antagonist program where an inverse agonist of the human H_4_-receptor was a partial agonist of rat and an antagonist of dog H_4_ receptor, complicating the interpretation of preclinical in vivo studies (Mowbray et al. 2011). Therefore, a greater appreciation for the structural basis for these effects could ultimately assist in prediction and systematic avoidance of similar issues in future programs.

Biased signalling is of current interest to both academia and the pharmaceutical industry based on the hypothesis that it could provide improved therapeutic benefit whilst avoiding undesirable activities that unbiased signalling of certain receptors has historically caused (Tautermann 2014). This has provided motivation to revisit some GPCR targets that were previously either poorly drugged or deemed undruggable due to lack of therapeutic index over adverse events (Correll and McKittrick 2014). However, despite advances in biology and chemistry providing assay methods to measure bias and clear examples of biased ligands emerging, a deeper structural understanding of protein conformational changes and interactions that lead to differential receptor signalling is still in its infancy but clearly of keen interest to the medicinal chemistry community (Violin et al. 2014).

**Fig. 9:**
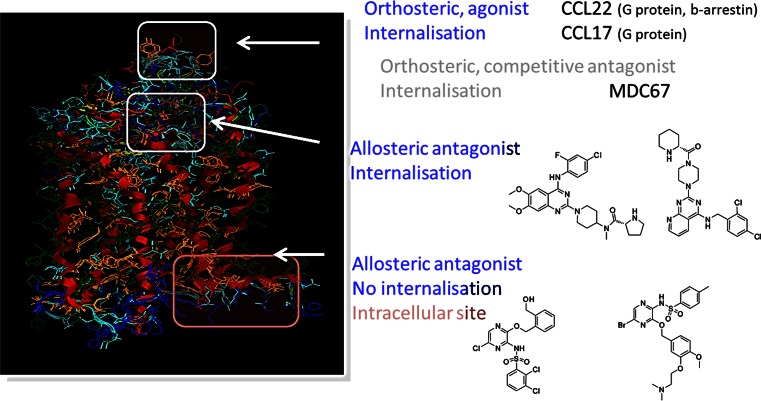
Homology model of the CCR_4_ receptor, with putative multiple binding sites for peptide agonists and small molecule allosteric ligands

Chemokine receptors are additional examples of ‘difficult’ GPCR targets, which usually have multiple peptide agonist ligands. CCR4 appears to have three different binding sites for peptide agonist and small molecule ligands (see Fig. 9). Complexity was further demonstrated in that two classes of chemically distinct small molecule ligands bind to different sites, both of which are allosteric modulators and also display different functional signalling (Procopiou et al. 2013; Slack et al. 2013; Ajram et al. 2014). Furthermore, one small molecule ligand site appears to be intracellular.

This complex picture of CCR_4_ resulted from extensive chemistry and pharmacological studies, and in hindsight it is clear why ‘traditional’ screening approaches such as binding or whole cell studies did not clearly identify the range of different ligands and sites. In one such screening approach, it was rationalised that the intracellular ligand binding site (helix 8) in CCR_4_ was likely modified owing the proximal chemical modification required in the commonly used FLIPR format.

**Fig. 10:**
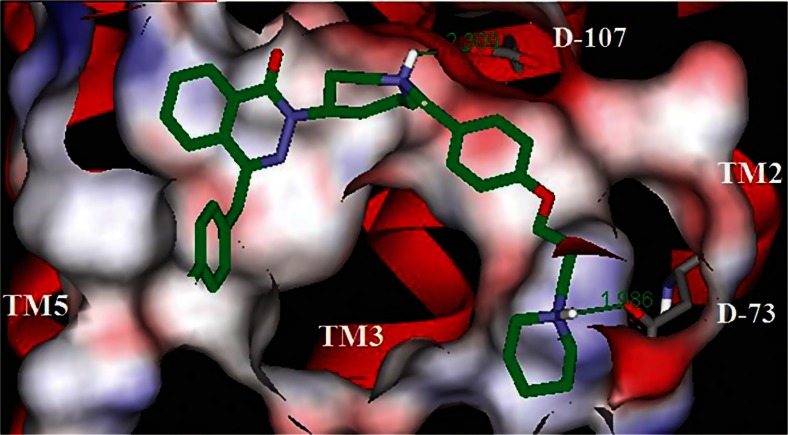
One of the low-energy conformations of the extended dual H_1_H_3_R ligands docked into the homology model of H1

Other examples of ‘difficult’ GPCR ligands include histamine dual H_1_R and H_3_R antagonists. Whilst the individual targets are well tractable individually, the design of single molecules that ‘fit’ and potently antagonise both receptors, and have added properties of broader selectivity and intranasal or oral drug properties, is extremely time consuming and challenging^.^ (Procopiou et al. 2011) (see Fig. 10).

Thus, structurally enabled approaches to ‘difficult’ GPRC targets should improve the tractability for chemistry by the direct identification of novel multiple ligand binding sites and subsequent chemical optimisation of small molecule ligands to drugs. This is an opportunity for drug discovery in a class of GPCR targets not easily accessible by previous methods.

Whilst many GPCR targets are chemically tractable resulting in multiple ligands and successful drugs, there are still many GPCR targets which are not amenable to ‘traditional’ approaches, such as focussed or high throughput screening, or where the natural ligands are unsuitable starting points for the design of oral drugs owing to their properties (e.g. large, lipophilic or peptidic).

## In silico driven GPCR drug discovery

Today, GPCR modelling is widely used in the structure-based drug discovery process. The availability of structural information on the binding site of a targeted GPCR plays a key role in rationalisation, efficiency and cost-effectiveness of drug design. X-ray crystallography, a traditional source of structural information, is not currently feasible for every GPCR or GPCR-ligand complex. This situation significantly limits the ability of crystallography to impact drug discovery for GPCR targets in ‘real-time’ and hence there is an urgent need for other practical alternatives. GPCR modelling is widely used as a practical alternative in the absence of crystallographic data, but can also provide much more useful information. Today, it can address such key issues like GPCR flexibility and dynamics, ligand kinetics (*k*_on_/*k*_off_ rates), prediction of water positions and their role in ligand binding, calculation of the free energy of binding (affinity) and prediction of the effects of mutations on ligand binding, etc. Some of these modelling approaches were reviewed in this meeting.

The role of flexibility and dynamics in the development of antipsychotic drugs and how this can be addressed by computational approaches was discussed. Recent pharmacological studies revealed that clinically effective antipsychotic agents act by binding to several bioamine receptors (Roth et al. 2004). In particular, the interaction with the serotonin (5-HT_2A_ and 5-HT_6_) and dopamine (D_2_ and D_3_) receptors (*group 1*) induces cognition-enhancing effects, while the histamine (H_1_), 5-HT_2C_ and 5-HT_2B_ receptors (group 2) modulation causes unwanted side effects (Selvam et al. 2013). Due to the complex pharmacological profile of CNS disorders, the attempts to develop drugs based on the one-target-one-disease paradigm have been limited (Allen and Roth 2011). As a result, there remains an urgent need for innovative approaches to develop new effective multi-target agents that improve patients’ health while reducing care costs. Ideal candidates should selectively target disease-active members of the family (*group 1*) while not binding to members responsible for undesired side effects (*group 2*). This is a very challenging goal to achieve, as the residues forming the orthosteric binding pocket, i.e. the binding site of endogenous ligands, are conserved within the receptor family, thus causing the recognition of a drug by many members. A strategy to overcome this issue is to design allosteric drugs targeting a less conserved allosteric site, which modulates the orthosteric site, or to design bitopic drugs, which bind to both allosteric and orthosteric sites.

**Fig. 11:**
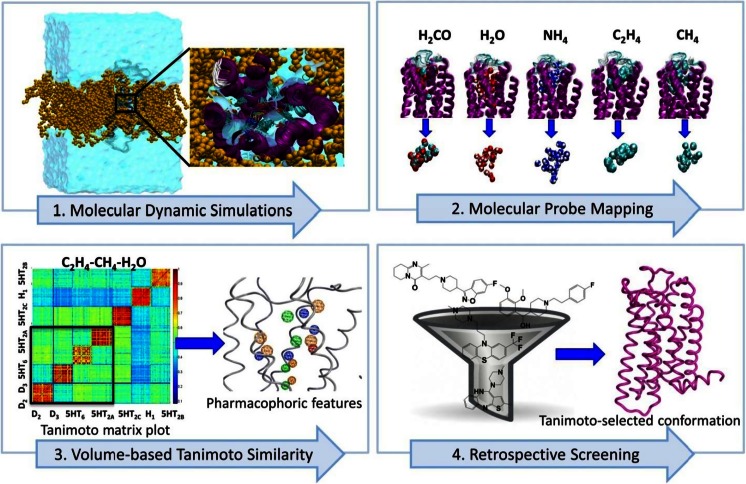
Structure-based computational protocol for selective polypharmacology—figure adapted from a recent publication (Selvam et al. 2013)

The recently released crystal structures of several bioamine receptors in complex with orthosteric and allosteric ligands enable the selectivity issue of antipsychotic drugs to be addressed at the molecular level. Thus, ‘structural snapshots’ provided by crystallography can be used to explore receptor motions using computer simulations. It is conceivable that allosteric and bitopic modulators interact with binding pockets that exist only in a subset of the receptor conformational space. Computer modelling can contribute to their identification by providing detailed insights into motions and interactions in the entire protein family and subsequently unravelling complex relationships in generated data within a reasonable time and at low cost. In academia, this approach has been undertaken with some promising results. For example, in the Tikhonova group, a computational protocol combining concepts from statistical mechanics and chemoinformatics have been developed to explore the flexibility of the bioamine receptors and identify geometrical and physicochemical properties that characterised the conformational space of the bioamine receptor family (Selvam et al. 2013). Figure 11 illustrates the molecular modelling steps undertaken to identify the unique pharmacophoric features of disease-active receptors.

The bioamine receptors of *groups 1* and *2* have been subjected to molecular dynamics simulations in a realistic environment. The results of simulations show substantial flexibility and its variability across the members of the receptor family. Using molecular probe mapping technique combined with the volume-based Tanimoto similarity measurements, similar and different geometrical and physicochemical properties were shown across the conformational space of the receptor family and the unique pharmacophoric features of disease-active receptors (group 1) were highlighted. The unique features are then linked with mutational and ligand structure-activity relationship data and tested in retrospective screening. The combination of techniques used gives an efficient method to identify unique properties of the disease-related proteins on the reduced diverse conformational space and represents a novel application of existing computational methods for the investigation of structural reasons for selective polypharmacology (Selvam et al. 2013). This protocol can be now exploited by industry for other protein families, involving in cancer and infectious diseases, which require a multi-target approach.

**Fig. 12:**
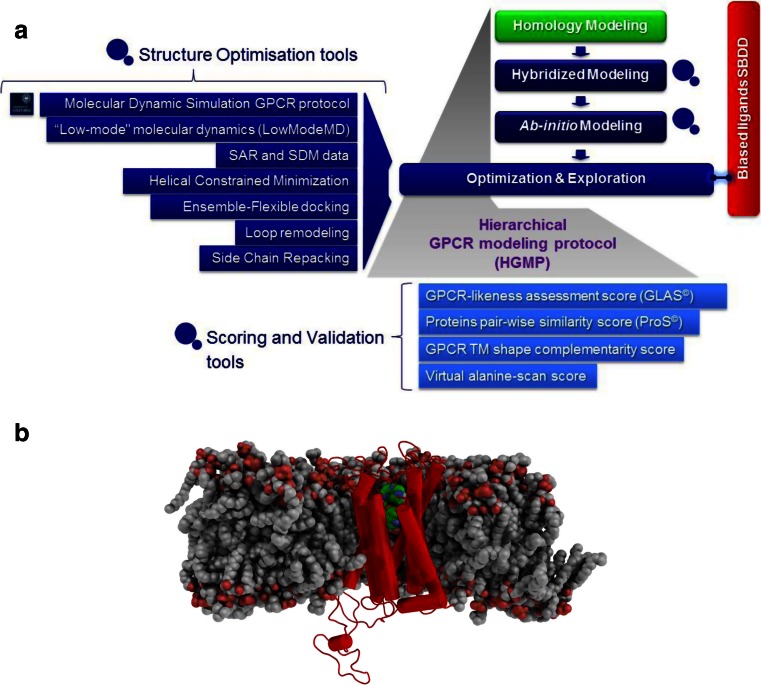
**a** HGMP workflow and **b** a model of 5-HT_2C_ (in *red*) produced by the HGMP workflow. The ligand is shown in *green* and the whole complex (Tye et al. 2011) is embedded in a membrane (*grey*). The water molecules and ions are omitted from the figure for clarity

In an industry setting, Evotec Ltd uses a hierarchical GPCR modelling protocol (HGMP) that has been developed in conjunction with the University of Oxford to support structure-based drug discovery programs (see Fig. 12a) (Heifetz et al. 2013a, b). The HGMP generates a 3D model of GPCR structures and its complexes with small molecules by applying a set of computational methods. The models produced by HGMP are then used in structure-based drug discovery. HGMP involves homology modelling, followed by MD simulation and flexible ensemble docking, to predict binding poses and function of ligands bound to GPCRs. The HGMP includes a large set of unique plugins to refine the GPCR models and exclusive scoring functions like the GPCR-likeness assessment score (GLAS) to evaluate model quality (Heifetz et al. 2013a). HGMP is also ‘armed’ with a pairwise protein comparison method (ProS) used to cluster the structural data generated by the HGMP and to distinguish between different activation sub-states. Recently, the capabilities of HGMP have been extended by the addition of GPCR biased ligand tools. The optimisation of HGMP has been performed by Evotec Ltd in real drug discovery projects.

The performance of HGMP in GPCR drug discovery projects such as MCH-1R for obesity treatment (Heifetz et al. 2013a), the Orexin-1 and -2 receptors for insomnia (Heifetz et al. 2012, 2013b) and the 5-HT_2C_ for the treatment of metabolic disorders (Tye et al. 2011; Storer et al. 2014) (see Fig. 12b) were highlighted. Additionally, the HGMP technology was used in the solving of the two H_1_R crystals structures bound to the second- and third-generation antihistamines: Cetirizine (Gillard et al. 2002) and Fexofenadine (Sharma et al. 2014), respectively, as described by Dr. Moraes (above).

Another area that has received increasing attention over recent years has been the role of water networks and their elucidation by MD simulations (Tautermann et al. 2015). With the availability of more very high-resolution GPCR structures [e.g. the δ-OR (Fenalti et al. 2014) or the A_2A_R (Liu et al. 2012) with resolutions of 1.8 Å], it has become clear that GPCRs often exhibit conserved water networks, which extend from the extracellular side to the intracellular surface. This conserved solvent network has been implied to be crucial for signalling (Nygaard et al. 2009) and a highly solvated conserved allosteric sodium binding site close to the conserved NPxxY motifs of class A GPCRs is postulated to be involved in β-arrestin signalling (Fenalti et al. 2014; Tautermann 2014). Beyond the functional effects, the consideration of water molecules in GPCR ligand design has been shown to be crucial (Bortolato et al. 2013) because several crystal structures show water-mediated ligand-protein interactions (Congreve et al. 2014). The displacement of binding site water upon ligand binding is energetically disadvantageous. Therefore, a ligand always has to gain more free energy from binding to the receptor than the removal of water actually costs. Several methods are available which enable the very crude and quick estimation of the energy penalty for water displacement, and most efficiently they are used for growing a bound ligand (Bortolato et al. 2013). They can help medicinal chemists to decide if a certain sub-pocket of the receptor can be explored by hydrophobic moieties or if a displaced water has to be substituted by an entity which has to mimic the hydrogen bond network. Obviously, these quick methods are not thorough in a sense that the estimates of the free energy are physically sound, but often they are sufficiently good for a go/no-go decision. In order to get a (formally) correct estimate for the change in the free energy of binding upon ligand modification, methods like thermodynamic integration have to be applied (Christ and Fox 2014). These more accurate methods all rely on long MD simulations of the receptor, and therefore they can also capture reorganisations in the solvent network and induced effects in the binding pocket. One step further beyond assessing the free energy of ligand binding is the estimation of binding kinetics of ligands. Water networks also play a crucial role here as well, as the breaking of ligand-receptor hydrogen bonds usually ends up with solvated hydrogen bond acceptor and donor functional groups. As the energy barrier of the reaction determines the rate constant, the direct interaction of water molecules with the ligand-protein hydrogen bonds is decisive for the dissociation rate. If the hydrogen bond is solvent accessible, the breaking of the bond does not require high energy because the re-hydration occurs simultaneously. If the hydrogen bond is buried, a breaking leads to a high energy penalty because acceptor and donor do not find immediate new interaction partners and are in unfavourable solvation states (Schmidtke et al. 2011). Currently, the in silico prediction of association/dissociation rates is only possible for very small fragments rather than drug-like molecules.

**Fig. 13:**
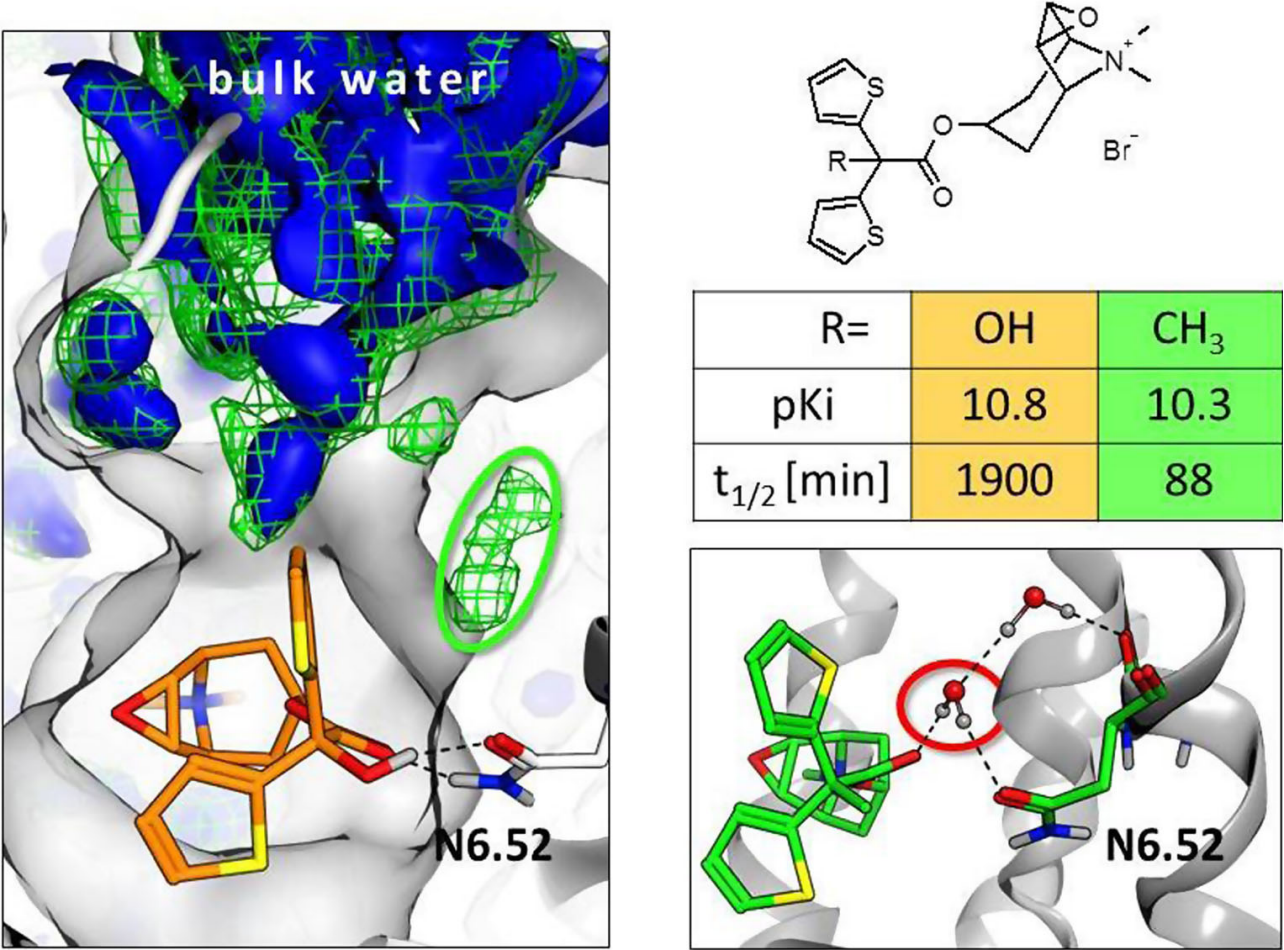
*Left*—comparison of the water (*oxygen*) densities in the tiotropium and the methyl ligand. The water densities of the tiotropium MD are displayed as *blue solid surfaces*; densities in methyl-ligand MDs are shown as *green mesh*. The significant extra density in the methyl-ligand MD is marked by a *green ellipse. Top right*—chemical structure of tiotropium (R=OH) and the methyl-ligand (R=CH_3_). *Middle right*—binding and dissociation constants of the ligands at the hM3R. *Bottom right*—a snapshot of the MD simulation with the methyl ligand, where water inserts into the ligand-protein hydrogen bond (in contrast to the tiotropium MD, where such water-mediated hydrogen bonds are never observed)—figure adapted from the recent publication (Tautermann et al. 2015)

Therefore, the state-of-the-art is still the explanation of experimental off-rates of drug-like molecules rather than the prediction of them. Recently, the duration of action of tiotropium on the human muscarinic M_3_ receptor (hM_3_R) was studied (Tautermann et al. 2013). Substitution of the hydroxy-group (see Fig. 13) by methyl does not reduce the p*K*_i_ strongly, but it has a major effect on the receptor half-life of the molecule. Long MD simulations (>2 μs) were used to investigate the differences in the bound species, especially focussing on the water network. Tiotropium forms a double hydrogen bond to N6.52 in hM3R (Fig. 13, left panel), and during the simulation no water comes close (blue solid surface). When substituting the hydroxy-group by methyl (‘methyl ligand’), water density is observed directly above the (single) hydrogen bond (green mesh), corresponding to a large number of MD snapshots where N6.52 is (partly) solvated. This comes along with a significantly widened exit channel of hM3R, and some MD snapshots even show water inserting in the hydrogen bond, as displayed in Fig. 13 (lower right panel). Thus, the explanation for the unexpected change in off-rates is the fact that the shielded hydrogen bond in bound tiotropium becomes solvent exposed when modifying the ligand. This observation was only possible through long MD simulations, and an explanation by the static x-ray structure would not have been possible. To summarise, the deep understanding of water networks within GPCRs has proven to be essential if one wants to understand GPCR signalling as well as GPCR ligand binding and dissociation. MD simulations are a very valuable tool to derive physically meaningful parameters such as free energy differences or solvent maps because they capture the dynamics of the systems, which is crucial especially for flexible proteins such as GPCRs.

An essential part of computational modelling is to re-evaluate predictions often in light of new structural data. Researchers at Novartis Horsham had performed a large amount of work exploring the binding sites of allosteric inhibitors of the chemokine receptor CXCR_2_, and several interesting things emerged after re-analysing this work in the light of crystallographic data (Salchow et al. 2010). It had been determined at Novartis Horsham that antagonists were acting at an allosteric binding site, and the patent literature around the time of the work suggested that the CXC chemokines could have a binding site close to the intracellular face of the receptor. One of the key residues in this binding site was proposed to be K320 as the CXCR_2_ antagonists commonly had an acidic functionality of some description within them and this residue was asparagine in the related CXCR_1_ receptor over which many of the CXCR_2_ antagonists had selectivity.

**Fig. 14:**
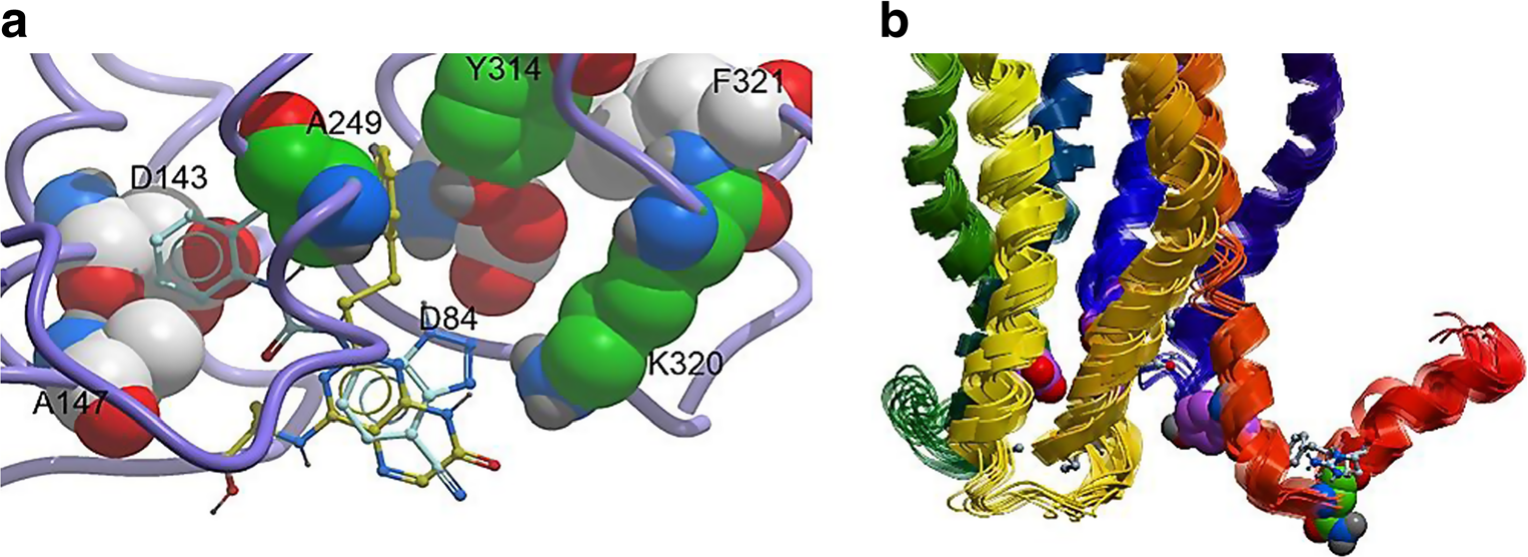
**a** The proposed binding modes for the quinoxaline (*yellow carbons*) and urea (*cyan carbons*) series with the influential mutated residues in CXCR_2_ shown in CPK. The consistent influence of K320 locates the acidic functionality in the antagonists, yet the varying effects of D143 on representatives of these two series suggest that the hydrophobic groups are located differently. **b** Overlay of the CXCR1 NMR structures from the PDB (code 2LNL) N320 is shown in *green* CPK and influential mutants from our experiments in *purple* CPK. The protein backbones, in ribbon representation, are coloured from N (*blue*) to C (*red*) termini

The mutagenesis experiments that were undertaken did show that residues at the intracellular end of the TM domains had an influence on the binding and/or potency of the antagonists whilst one proposed as critical within the TM domain did not show any influence. However, the expected overlay of the antagonists based upon their ligand-only overlays was not reproduced in the effects seen against the various mutant receptors. The presumed binding modes derived from this work are shown in Fig. 14a. The residues proposed for mutagenesis, and the interpretation of their effects, were very dependent upon the model used to create the homology model for CXCR_2_ at the time, and so a review of how the latest GPCR crystal structures could have influenced this project was presented.

The NMR structures for the related CXCR_1_ receptor (as reproduced in Fig. 14b) consistently show that the critical N320 residue is not close to the other influential residues, and it would have been hard to rationalise the mutagenesis from this template. The CCR_5_ crystal structure shows distortion in the TM7/helix 8 region and the critical K320 residue was mutated to a GLU, but overall the helical alignment compared to Rhodopsin was very similar and so would not have provided any benefit compared to the Rhodopsin template used originally. The β_2_AR-G_s_-protein structure was a marvellous achievement (Rasmussen et al. 2011), but for the purposes of acting as a template for the intracellular binding area of the antagonists, it is likely to be too different to CXCR_2_ to be useful. The most relevant structure could have been the CXCR_4_, but here again the TM7/helix 8 region is distorted in the crystal and a CXCR_2_ homology model was created by using a chimeric template of this receptor and the TM7/helix 8 region from an earlier β_2_AR structure (Rasmussen et al. 2011). Interestingly, this model had a related CXCR_2_ antagonist bound in the TM domain and the major learning point from the current GPCR symposium was just how much influence changes within the TM domain affected the intracellular coupling and vice versa. A conclusion drawn from the symposium with respect to the work completed at NIBR Horsham would be that more mutagenesis information from across the whole of the CXCR_2_ protein would have been needed in order to fully understand the binding area of these allosteric antagonists.

Finally, network-based approaches, evolutionary algorithms and predictive modelling, all areas that will become more prevalent in the future, were discussed. The advent of the ‘omics’ age has brought with it huge quantities of data around diseases, targets, compounds and their effects. Networks of interactions and disease ‘interactomes’ can be built with the ultimate goal to understand disease networks and how they are influenced by the changes in small molecules and their properties.

**Fig. 15:**
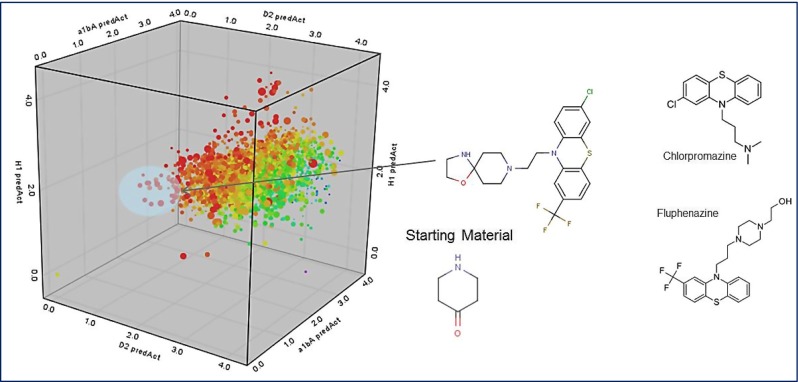
Automated multi-objective compound design using reaction vectors (26K Reaction Db and 93K Reagents) starting from piperidine and using four objectives: similarity to haloperidol and Ziprasidone pharmacophores, Dopamine D_2_, α1B Adrenergic and Histamine QSAR models. The tri-cyclics generated appeared similar to known anti-pyschotics, Chlorpromazine and Fluphenazine

The polypharmacology associated with current typical and atypical anti-psychotics is complex, and as an example, the question of how do we go about designing a novel anti-psychotic given the tools and data we have access to today was raised. The opensource ChEMBL space polypharmacology network viewer (Fechner et al. 2013) was introduced as an interactive way to review the rich pharmacology accessible in the ChEMBL database and identify some good starting points for drug design. The experimental polypharmacology associated with the hits can be complemented using target prediction ligand similarity-based approaches such as the similarity ensemble approach (SEA; Keiser et al. 2009) or broad panel-based predictive modelling approaches (Ghosh and Jones 2014). Predictive modelling approaches were also used to build protein target QSARs that in combination with pharmacophore triplet compound similarity were used to develop a multi-objective scoring function. Given a small organic fragment, an automated evolutionary design algorithm using reaction vectors was used to grow a molecule by simultaneously optimising the multi-parameters required for the targeted phenotype polypharmacology (Patel et al. 2009) (see Fig. 15). The reaction vector design approach was extended to whole reaction sequences and ultimately reaction networks. A GPR38 reaction network was built which exemplified that the chemistry phase space around a hit could be readily expanded to that of closely accessible molecules. This would enable better sampling and rapid medchem design.

It was also shown how GPCR targets and their interacting partners could be identified from Genome Wide Association Studies (GWAS) using network analysis followed by the analysis of compound gene expression data to complement target disease gene expression as a strategy for network-based drug design. Multiple algorithms have been developed that in combination with omics data and GPCR structure-based design make a powerful arsenal for today’s drug designer.

## Conclusions

All participants agreed that GPCR research and drug discovery can benefit greatly from the collaboration between academia and industry. The effectiveness of such collaborations for GPCR research and drug discovery was widely described and exemplified during this conference: the non-profit GPCR Consortium (http://gpcrconsortium.org) described by Vadim Cherezov; the collaboration between MRC Laboratory and Heptares mentioned by Gebhard Schertler and Chris Tate; the consortium between Evotec Ltd, Oxford University UK and the Membrane Protein Laboratory at Diamond Light Source, UK described by Alexander Heifetz; and many other successful networks like the Adhesion-GPCR Consortium (AGC) or GLISTEN (Gutierrez-de-Teran 2014) (GPCR-Ligand Interactions, Structures, and Transmembrane Signalling: a European Research Network).

There was broad agreement with Schertler’s and Ceska’s comments that most ventures between academia and industry are dependent on track record and trust and that individuals have to commit to a longer-term perspective which is aimed at changing the scientific landscape. There was also agreement that meetings between academia and industry such as this conference are very useful to learn about developments within each other’s areas of expertise and to share the challenges whilst forging new links and networks. For future meetings of this type, it was proposed to also include a broader mix of younger-generation scientists early in their career in order to maximise benefit to a broader community.

## References

[CR1] Ajram L, Begg M, Slack R, Cryan J, Hall D, Hodgson S, Ford A, Barnes A, Swieboda D, Mousnier A, Solari R (2014). Internalization of the chemokine receptor CCR4 can be evoked by orthosteric and allosteric receptor antagonists. Eur J Pharmacol.

[CR2] Allen JA, Roth BL (2011). Strategies to discover unexpected targets for drugs active at G protein-coupled receptors. Annu Rev Pharmacol Toxicol.

[CR3] Bennett KA, Dore AS, Christopher JA, Weiss DR, Marshall FH (2014). Structures of mGluRs shed light on the challenges of drug development of allosteric modulators. Curr Opin Pharmacol.

[CR4] Bortolato A, Tehan BG, Bodnarchuk MS, Essex JW, Mason JS (2013). Water network perturbation in ligand binding: adenosine A(2A) antagonists as a case study. J Chem Inf Model.

[CR5] Breitman M, Kook S, Gimenez LE, Lizama BN, Palazzo MC, Gurevich EV, Gurevich VV (2012). Silent scaffolds: inhibition of JNK3 activity in the cell by a dominant-negative arrestin-3 mutant. J Biol Chem.

[CR6] Canonica GW, Blaiss M (2011). Antihistaminic, anti-inflammatory, and antiallergic properties of the nonsedating second-generation antihistamine desloratadine: a review of the evidence. World Allergy Organ J.

[CR7] Christ CD, Fox T (2014). Accuracy assessment and automation of free energy calculations for drug design. J Chem Inf Model.

[CR8] Christopher JA, Brown J, Dore AS, Errey JC, Koglin M, Marshall FH, Myszka DG, Rich RL, Tate CG, Tehan B, Warne T, Congreve M (2013). Biophysical fragment screening of the beta1-adrenergic receptor: identification of high affinity arylpiperazine leads using structure-based drug design. J Med Chem.

[CR9] Congreve M, Dias JM, Marshall FH (2014). Structure-based drug design for G protein-coupled receptors. Prog Med Chem.

[CR10] Correll CC, McKittrick BA (2014). Biased ligand modulation of seven transmembrane receptors (7TMRs): functional implications for drug discovery. J Med Chem.

[CR11] Fechner N, Papadatos G, Evans D, Morphy JR, Brewerton SC, Thorner D, Bodkin M (2013). ChEMBLSpace—a graphical explorer of the chemogenomic space covered by the ChEMBL database. Bioinformatics.

[CR12] Fenalti G, Giguere PM, Katritch V, Huang XP, Thompson AA, Cherezov V, Roth BL, Stevens RC (2014). Molecular control of delta-opioid receptor signalling. Nature.

[CR13] Ghosh B, Jones LH (2014). Target validation using in-cell small molecule clickable imaging probes. MedChemComm.

[CR14] Gillard M, Van Der Perren C, Moguilevsky N, Massingham R, Chatelain P (2002). Binding characteristics of cetirizine and levocetirizine to human H(1) histamine receptors: contribution of Lys(191) and Thr(194). Mol Pharmacol.

[CR15] Gimenez LE, Vishnivetskiy SA, Baameur F, Gurevich VV (2012). Manipulation of very few receptor discriminator residues greatly enhances receptor specificity of non-visual arrestins. J Biol Chem.

[CR16] Gurevich EV, Gurevich VV (2006). Arrestins are ubiquitous regulators of cellular signaling pathways. Genome Biol.

[CR17] Gurevich EV, Gurevich VV (2014). Therapeutic potential of small molecules and engineered proteins. Handb Exp Pharmacol.

[CR18] Gurevich VV, Gurevich EV (2012). Synthetic biology with surgical precision: targeted reengineering of signaling proteins. Cell Signal.

[CR19] Gurevich VV, Gurevich EV (2014). Extensive shape shifting underlies functional versatility of arrestins. Curr Opin Cell Biol.

[CR20] Gutierrez-de-Teran H (2014). The roles of computational chemistry in the ligand design of G protein-coupled receptors: how far have we come and what should we expect?. Future Med Chem.

[CR21] Heifetz A, Barker O, Morris GB, Law RJ, Slack M, Biggin PC (2013). Toward an understanding of agonist binding to human Orexin-1 and Orexin-2 receptors with G-protein-coupled receptor modeling and site-directed mutagenesis. Biochemistry.

[CR22] Heifetz A, Barker O, Verquin G, Wimmer N, Meutermans W, Pal S, Law RJ, Whittaker M (2013). Fighting obesity with a sugar-based library: discovery of novel MCH-1R antagonists by a new computational-VAST approach for exploration of GPCR binding sites. J Chem Inf Model.

[CR23] Heifetz A, Morris GB, Biggin PC, Barker O, Fryatt T, Bentley J, Hallett D, Manikowski D, Pal S, Reifegerste R, Slack M, Law R (2012). Study of human Orexin-1 and -2G-protein-coupled receptors with novel and published antagonists by modeling, molecular dynamics simulations, and site-directed mutagenesis. Biochemistry.

[CR24] Keiser MJ, Setola V, Irwin JJ, Laggner C, Abbas AI, Hufeisen SJ, Jensen NH, Kuijer MB, Matos RC, Tran TB, Whaley R, Glennon RA, Hert J, Thomas KL, Edwards DD, Shoichet BK, Roth BL (2009). Predicting new molecular targets for known drugs. Nature.

[CR25] Koole C, Pabreja K, Savage EE, Wootten D, Furness SG, Miller LJ, Christopoulos A, Sexton PM (2013). Recent advances in understanding GLP-1R (glucagon-like peptide-1 receptor) function. Biochem Soc Trans.

[CR26] Koole C, Savage EE, Christopoulos A, Miller LJ, Sexton PM, Wootten D (2013). Minireview: signal bias, allosterism, and polymorphic variation at the GLP-1R: implications for drug discovery. Mol Endocrinol.

[CR27] Kruse AC, Hu J, Pan AC, Arlow DH, Rosenbaum DM, Rosemond E, Green HF, Liu T, Chae PS, Dror RO, Shaw DE, Weis WI, Wess J, Kobilka BK (2012). Structure and dynamics of the M3 muscarinic acetylcholine receptor. Nature.

[CR28] Liu W, Chun E, Thompson AA, Chubukov P, Xu F, Katritch V, Han GW, Roth CB, Heitman LH, IJzerman AP, Cherezov V, Stevens RC (2012). Structural basis for allosteric regulation of GPCRs by sodium ions. Science.

[CR29] Liu W, Ishchenko A, Cherezov V (2014). Preparation of microcrystals in lipidic cubic phase for serial femtosecond crystallography. Nat Protoc.

[CR30] Liu W, Wacker D, Gati C, Han GW, James D, Wang D, Nelson G, Weierstall U, Katritch V, Barty A, Zatsepin NA, Li D, Messerschmidt M, Boutet S, Williams GJ, Koglin JE, Seibert MM, Wang C, Shah ST, Basu S, Fromme R, Kupitz C, Rendek KN, Grotjohann I, Fromme P, Kirian RA, Beyerlein KR, White TA, Chapman HN, Caffrey M, Spence JC, Stevens RC, Cherezov V (2013). Serial femtosecond crystallography of G protein-coupled receptors. Science.

[CR31] Liu W, Wacker D, Wang C, Abola E, Cherezov V (2014). Femtosecond crystallography of membrane proteins in the lipidic cubic phase. Philos Trans R Soc Lond B Biol Sci.

[CR32] Magnan R, Escrieut C, Gigoux V, De K, Clerc P, Niu F, Azema J, Masri B, Cordomi A, Baltas M, Tikhonova IG, Fourmy D (2013). Distinct CCK-2 receptor conformations associated with beta-arrestin-2 recruitment or phospholipase-C activation revealed by a biased antagonist. J Am Chem Soc.

[CR33] Magnan R, Masri B, Escrieut C, Foucaud M, Cordelier P, Fourmy D (2011). Regulation of membrane cholecystokinin-2 receptor by agonists enables classification of partial agonists as biased agonists. J Biol Chem.

[CR34] Michel MC, Seifert R, Bond RA (2014). Dynamic bias and its implications for GPCR drug discovery. Nat Rev Drug Discov.

[CR35] Miller-Gallacher JL, Nehme R, Warne T, Edwards PC, Schertler GF, Leslie AG, Tate CG (2014). The 2.1 A resolution structure of cyanopindolol-bound beta1-adrenoceptor identifies an intramembrane Na+ ion that stabilises the ligand-free receptor. PLoS One.

[CR36] Monck NJ, Kennett GA (2008). 5-HT2C ligands: recent progress. Prog Med Chem.

[CR37] Mowbray CE, Bell AS, Clarke NP, Collins M, Jones RM, Lane CA, Liu WL, Newman SD, Paradowski M, Schenck EJ, Selby MD, Swain NA, Williams DH (2011). Challenges of drug discovery in novel target space. The discovery and evaluation of PF-3893787: a novel histamine H4 receptor antagonist. Bioorg Med Chem Lett.

[CR38] Nygaard R, Frimurer TM, Holst B, Rosenkilde MM, Schwartz TW (2009). Ligand binding and micro-switches in 7TM receptor structures. Trends Pharmacol Sci.

[CR39] Oppenheimer JJ, Casale TB (2002). Next generation antihistamines: therapeutic rationale, accomplishments and advances. Expert Opin Investig Drugs.

[CR40] Patel H, Bodkin MJ, Chen B, Gillet VJ (2009). Knowledge-based approach to de novo design using reaction vectors. J Chem Inf Model.

[CR41] Procopiou PA, Barrett JW, Barton NP, Begg M, Clapham D, Copley RC, Ford AJ, Graves RH, Hall DA, Hancock AP, Hill AP, Hobbs H, Hodgson ST, Jumeaux C, Lacroix YM, Miah AH, Morriss KM, Needham D, Sheriff EB, Slack RJ, Smith CE, Sollis SL, Staton H (2013). Synthesis and structure-activity relationships of indazole arylsulfonamides as allosteric CC-chemokine receptor 4 (CCR4) antagonists. J Med Chem.

[CR42] Procopiou PA, Browning C, Buckley JM, Clark KL, Fechner L, Gore PM, Hancock AP, Hodgson ST, Holmes DS, Kranz M, Looker BE, Morriss KM, Parton DL, Russell LJ, Slack RJ, Sollis SL, Vile S, Watts CJ (2011). The discovery of phthalazinone-based human H1 and H3 single-ligand antagonists suitable for intranasal administration for the treatment of allergic rhinitis. J Med Chem.

[CR43] Qin X (2007). What caused the increase of autoimmune and allergic diseases: a decreased or an increased exposure to luminal microbial components?. World J Gastroenterol.

[CR44] Rasmussen SG, DeVree BT, Zou Y, Kruse AC, Chung KY, Kobilka TS, Thian FS, Chae PS, Pardon E, Calinski D, Mathiesen JM, Shah ST, Lyons JA, Caffrey M, Gellman SH, Steyaert J, Skiniotis G, Weis WI, Sunahara RK, Kobilka BK (2011). Crystal structure of the beta2 adrenergic receptor-Gs protein complex. Nature.

[CR45] Reinartz MT, Kalble S, Littmann T, Ozawa T, Dove S, Kaever V, Wainer IW, Seifert R (2015). Structure-bias relationships for fenoterol stereoisomers in six molecular and cellular assays at the beta-adrenoceptor. Naunyn Schmiedebergs Arch Pharmacol 388(1):51–65 10.1007/s00210-014-1054-5 25342094

[CR46] Roth BL, Sheffler DJ, Kroeze WK (2004). Magic shotguns versus magic bullets: selectively non-selective drugs for mood disorders and schizophrenia. Nat Rev Drug Discov.

[CR47] Salchow K, Bond ME, Evans SC, Press NJ, Charlton SJ, Hunt PA, Bradley ME (2010). A common intracellular allosteric binding site for antagonists of the CXCR2 receptor. Br J Pharmacol.

[CR48] Schmidtke P, Luque FJ, Murray JB, Barril X (2011). Shielded hydrogen bonds as structural determinants of binding kinetics: application in drug design. J Am Chem Soc.

[CR49] Schöneberg T, Schulz A, Biebermann H, Hermsdorf T, Römpler H, Sangkuhl K (2004). Mutant G-protein-coupled receptors as a cause of human diseases. Pharmacol Ther.

[CR50] Seifert R (2013). Functional selectivity of G-protein-coupled receptors: from recombinant systems to native human cells. Biochem Pharmacol.

[CR51] Seifert R, Dove S (2009). Functional selectivity of GPCR ligand stereoisomers: new pharmacological opportunities. Mol Pharmacol.

[CR52] Selvam B, Porter SL, Tikhonova IG (2013). Addressing selective polypharmacology of antipsychotic drugs targeting the bioaminergic receptors through receptor dynamic conformational ensembles. J Chem Inf Model.

[CR53] Sharma M, Bennett C, Cohen SN, Carter B (2014). H1-antihistamines for chronic spontaneous urticaria. Cochrane Database Syst Rev.

[CR54] Shibata Y, Gvozdenovic-Jeremic J, Love J, Kloss B, White JF, Grisshammer R, Tate CG (2013). Optimising the combination of thermostabilising mutations in the neurotensin receptor for structure determination. Biochim Biophys Acta.

[CR55] Shimamura T, Shiroishi M, Weyand S, Tsujimoto H, Winter G, Katritch V, Abagyan R, Cherezov V, Liu W, Han GW, Kobayashi T, Stevens RC, Iwata S (2011). Structure of the human histamine H1 receptor complex with doxepin. Nature.

[CR56] Simons FE, Simons KJ (2011) Histamine and H1-antihistamines: celebrating a century of progress. J Allergy Clin Immunol 128(6): 1139–1150.e1134 10.1016/j.jaci.2011.09.005 22035879

[CR57] Slack RJ, Russell LJ, Barton NP, Weston C, Nalesso G, Thompson SA, Allen M, Chen YH, Barnes A, Hodgson ST, Hall DA (2013). Antagonism of human CC-chemokine receptor 4 can be achieved through three distinct binding sites on the receptor. Pharmacol Res Perspect.

[CR58] Song X, Vishnivetskiy SA, Gross OP, Emelianoff K, Mendez A, Chen J, Gurevich EV, Burns ME, Gurevich VV (2009). Enhanced arrestin facilitates recovery and protects rod photoreceptors deficient in rhodopsin phosphorylation. Curr Biol.

[CR59] Storer RI, Brennan PE, Brown AD, Bungay PJ, Conlon KM, Corbett MS, DePianta RP, Fish PV, Heifetz A, Ho DK, Jessiman AS, McMurray G, de Oliveira CA, Roberts LR, Root JA, Shanmugasundaram V, Shapiro MJ, Skerten M, Westbrook D, Wheeler S, Whitlock GA, Wright J (2014). Multiparameter optimization in CNS drug discovery: design of pyrimido[4,5-d]azepines as potent 5-hydroxytryptamine 2C (5-HT(2)C) receptor agonists with exquisite functional selectivity over 5-HT(2)A and 5-HT(2)B receptors. J Med Chem.

[CR60] Tan Q, Zhu Y, Li J, Chen Z, Han GW, Kufareva I, Li T, Ma L, Fenalti G, Li J, Zhang W, Xie X, Yang H, Jiang H, Cherezov V, Liu H, Stevens RC, Zhao Q, Wu B (2013). Structure of the CCR5 chemokine receptor-HIV entry inhibitor maraviroc complex. Science.

[CR61] Tate CG (2012). A crystal clear solution for determining G-protein-coupled receptor structures. Trends Biochem Sci.

[CR62] Tautermann CS (2014). GPCR structures in drug design, emerging opportunities with new structures. Bioorg Med Chem Lett.

[CR63] Tautermann CS, Kiechle T, Seeliger D, Diehl S, Wex E, Banholzer R, Gantner F, Pieper MP, Casarosa P (2013). Molecular basis for the long duration of action and kinetic selectivity of tiotropium for the muscarinic M3 receptor. J Med Chem.

[CR64] Tautermann CS, Seeliger D, Kriegl JM (2015). What can we learn from molecular dynamics simulations for GPCR drug design?. Comput Struct Biotechnol J.

[CR65] Tye H, Mueller SG, Prestle J, Scheuerer S, Schindler M, Nosse B, Prevost N, Brown CJ, Heifetz A, Moeller C, Pedret-Dunn A, Whittaker M (2011). Novel 6,7,8,9-tetrahydro-5H-1,4,7,10a-tetraaza-cyclohepta[f]indene analogues as potent and selective 5-HT(2C) agonists for the treatment of metabolic disorders. Bioorg Med Chem Lett.

[CR66] Violin JD, Crombie AL, Soergel DG, Lark MW (2014). Biased ligands at G-protein-coupled receptors: promise and progress. Trends Pharmacol Sci.

[CR67] Vishnivetskiy SA, Chen Q, Palazzo MC, Brooks EK, Altenbach C, Iverson TM, Hubbell WL, Gurevich VV (2013). Engineering visual arrestin-1 with special functional characteristics. J Biol Chem.

[CR68] Vishnivetskiy SA, Gimenez LE, Francis DJ, Hanson SM, Hubbell WL, Klug CS, Gurevich VV (2011). Few residues within an extensive binding interface drive receptor interaction and determine the specificity of arrestin proteins. J Biol Chem.

[CR69] Weierstall U, James D, Wang C, White TA, Wang D, Liu W, Spence JC, Bruce Doak R, Nelson G, Fromme P, Fromme R, Grotjohann I, Kupitz C, Zatsepin NA, Liu H, Basu S, Wacker D, Han GW, Katritch V, Boutet S, Messerschmidt M, Williams GJ, Koglin JE, Marvin Seibert M, Klinker M, Gati C, Shoeman RL, Barty A, Chapman HN, Kirian RA, Beyerlein KR, Stevens RC, Li D, Shah ST, Howe N, Caffrey M, Cherezov V (2014). Lipidic cubic phase injector facilitates membrane protein serial femtosecond crystallography. Nat Commun.

[CR70] Wootten D, Savage EE, Willard FS, Bueno AB, Sloop KW, Christopoulos A, Sexton PM (2013). Differential activation and modulation of the glucagon-like peptide-1 receptor by small molecule ligands. Mol Pharmacol.

[CR71] Wootten D, Simms J, Miller LJ, Christopoulos A, Sexton PM (2013). Polar transmembrane interactions drive formation of ligand-specific and signal pathway-biased family B G protein-coupled receptor conformations. Proc Natl Acad Sci U S A.

[CR72] Zhan X, Perez A, Gimenez LE, Vishnivetskiy SA, Gurevich VV (2014). Arrestin-3 binds the MAP kinase JNK3α2 via multiple sites on both domains. Cell Signal.

